# Female career advancement in obstetrics/gynecology, and urology: a spatio-temporal comparative study over the last 2 decades in Germany

**DOI:** 10.1007/s00404-026-08511-2

**Published:** 2026-09-03

**Authors:** Lena Löffler, Lisa Jörgens, Stefanie Mache, Daniela Ohlendorf, Doris Klingelhöfer, Koji Matsuo, Jenny Jaque, Isabelle Marie Kramer, Dörthe Brüggmann

**Affiliations:** 1 Institute of Occupational, Social and Environmental Medicine, Goethe-University Frankfurt, Frankfurt, Germany; 2 Institute for Occupational and Maritime Medicine (ZfAM), University Medical Center Hamburg-Eppendorf (UKE), Hamburg, Germany; 3 Division of Gynecologic Oncology, Department of Obstetrics and Gynecology, University of Southern California, Los Angeles, CA USA; 4 Department of Obstetricsand Gynecology, Los Angeles General Medical Center, Los Angeles, CA USA; 5 Norris Comprehensive Cancer Center, University of Southern California, Los Angeles, CA USA

**Keywords:** Gender, Equity, Index, Career promotion, Academics, Medicine

## Abstract

**Introduction:**

Most medical graduates and specialists of obstetrics and gynecology (OB/GYN) in Germany are female, and numbers of female urology specialists are rising. However, the number of women in higher academic positions does not correlate with this increase. This study analyzes female academic career advancement on a spatial and temporal scale in Germany using the Brüggmann–Groneberg (BG) Index. The academic BG Index relates female-to-male (f:m) ratios in chair positions of state universities while representing lower career levels. It is updated in this study from 2013 (OB/GYN) and 2015 (urology) onwards, while a modified clinical BG (BGclin) Index, introduced here for the first time, assesses career progression ratios in leadership positions in clinical medicine.

**Methods:**

The gender distribution among medical students, OB/GYN and urology specialists, clinical leaders, and academic chair holders was obtained from governmental and non-governmental sources. The f:m ratios of the populations were assessed and used in the BG indices which were calculated for five time points (2004, 2009, 2014, 2019 and 2024) and across Germany to identify spatial–temporal trends.

**Results:**

F:m ratios among hospital specialists increased in OB/GYN (0.847 to 2.388) and urology (0.13 to 0.361) from 2004 to 2024. In contrast to urology, significant changes in the distribution of male and female chair holders toward gender equality could be observed in OB/GYN. Simultaneously, the academic BG Index rose from 0.045 to 0.099 in OB/GYN and from 0 to 0.084 in urology. The BGclin Index slightly decreased in OB/GYN (0.108 to 0.102) and increased in urology (0.086 to 0.114). No spatial trend could be observed.

**Discussion:**

This study demonstrates persistent gender imbalance in German academic and clinical leadership positions in OB/GYN and urology. The academic and BGclin Indices indicate stagnation and/or decline in gender parity, emphasizing the need for targeted career development programs. Furthermore, a spatial analysis revealed an absence of spatial patterns in German chair holder positions. The continued use of these indices will prove a helpful tool to assess and document changes in the upcoming years.

**Supplementary Information:**

The online version contains supplementary material available at https://doi.org/10.1007/s00404-026-08511-2.

## What does this study add to the clinical work?

**Table:** 

This study analyzes female academic career advancement of OB/GYN and urology on a spatial and temporal scale in Germany using the known (academic) Brüggmann–Groneberg (BG) Index and introducing for the first time the modified, clinical BG Index, hence assessing career progression ratios in top and leadership positions, while taking lower career positions into account. From 2004 to 2024, we demonstrate stagnation or in some cases decline in gender parity, while also revealing an absence of spatial patterns in German chair holder positions, thus underlining the need for targeted career development programs, whose success can be assessed by the continued used of these indices.	

## Introduction

After Elizabeth Blackwell became the first woman to receive a medical degree in 1849 in the Western world [1, 2], the number of female medical students and practitioners rose drastically over the years [3]. In Germany, the turning point was in 1999 when, for the first time, more women than men started studying medicine [4]. In the coming years, the ratio of female-to-male medical students continued to rise; however, the amount of women in higher academic positions does not correlate with this increase [5]. In Germany, the difference between the percentage of female senior physicians and female chairs is eminent [6]. While 69% of all senior physicians in Germany are female, only 25% hold the position of the academic chair [6]. The reasons are multifaceted, ranging from gender disparity in appointment committees with the crucial group composed of a high percentage of men [7], to a lack of formal and informal mentoring [8]. Research discussing gender bias in academic medicine shows divergencies in different medical fields [6, 9, 10]. A recent study by Neuhold and von Versen-Höynck conducted a cross-sectional analysis using a database from the German Society for Gynecology and Obstetrics (DGGG) [11]. They concluded that while gender diversity in the clinical workforce has improved, systemic inequities persist in academic and leadership domains [11]. We focused on academic leadership positions and demonstrated the persistence of leadership inequity in obstetrics and gynecology (OB/GYN) and urology. One tool to assess potential disparities in career advancement is the academic Brüggmann–Groneberg (BG) Index that was first described in 2017 and reported by Nature to be useful in analyzing female-to-male (f:m) ratios in higher academic positions [10, 12]. It describes the balance between female and male physicians in the position of the academic chair, while considering the lower academic positions. An equilibrium of female and male academic chair holders would be indicated with the academic BG Index being 1.0.

The academic BG Index was first used to analyze and compare the fields of OB/GYN and ears, nose, and throat medicine (ENT) in 2017. This research continued with the field of urology in 2020, comparing it to the previously determined academic BG Index of OB/GYN and ENT, showing greater opportunities of career promotion for female physicians in the field of urology at the time of comparison [9].

This study aims to bring the previously determined academic BG Index of OB/GYN and urology up to date, analyzing the development of female career promotion aspects. Moreover, this study introduces the BGclin Index for the previously mentioned fields, which analyzes the gender disparities within leadership positions (chief physicians) in clinical medicine, as it focuses on the gender of the physicians in leadership positions as opposed to the academic chair holders, to determine female career aspects in clinical medicine. Taken together, this study aims to analyze the f:m ratio as well as the academic and BGclin Index from 2004 to 2024 over time, including spatial differences across Germany, since the historical division of Germany until 1989 with the result of a substantial migration of 10% of East German citizens, predominantly young women, to West Germany may have led to differences of gender parity in professional settings [13].

## Methods

### Rationale for the selection of the two comparator specialties

Since the use of the BG Index in 2020 revealed greater opportunities of career promotion for female physicians in the field of urology in comparison to OB/GYN and ENT [10], we determined to compare urology and OB/GYN to ensure continuity, while also gaining new insights by introducing a new index.

### Data collection and calculation of sex ratio

We assessed the quantity of female and male medical students, practitioners, physicians in leadership positions and academic chair holders/full professors for the years 2004, 2009, 2014, 2019 and 2024 across Germany for the specialties of OB/GYN and urology. As previously described [10], the number of medical students was supplied by the DeSTATIS database, a federal statistics database by the Federal Ministry of Internal Affairs which is updated yearly [4, 10]. The yearly updated numbers of female and male specialized hospital physicians, as well as physicians in leadership positions, were provided by the German Medical Association [3]. Leadership positions are denoted by chief physicians and do not include managing physicians. The last required set of data was the proportion of men and women in the highest academic position, defined as the head of the department in a university hospital and academic chair holder/full professor in the respective field. The number of female and male physicians in this position was determined for all (38 in 2004, and 43 by 2024) state universities by screening the respective websites, consultations of journals, and written or oral enquiry with the departments or dean’s offices. The gender of the academic chair holder was determined as follows. First, name-to-gender assignment was performed via https://genderize.io/. In case of certainty below 99%, gender was determined by either form of address (Mr. or Ms.) and used pronouns in case of consultation of written sources, or by direct enquiry with the departments or dean’s offices. Via these methods, every person could be assigned to a gender.

Ethical considerations were addressed by consulting the ethics committee of the medical department of the University of Frankfurt, which concluded that the methods for data collection used in this study do not need further assessment by the ethics committee.

### Calculation of the sex ratio

To calculate the BG Index, the f:m ratios of each career position and reference year were determined. The f:m ratio was used in this study, since the calculation is easiest to reproduce by future studies with the dataset provided by the DeSTATIS database and German Medical Association. To calculate the f:m ratio, the number of female medical students was divided by the number of male medical students for each of the 5 years, the number of female practitioners in the respective field in a hospital setting was divided by the number of male practitioners in the same position, and the number of female academic chair holders was divided by the number of male academic chair holders in the corresponding field. In the event of a change of the academic chair holder during an analyzed year the gender of the last chair holder/full professor was acknowledged and taken into consideration in the calculation. Provisional academic chair holders were not considered.

The previously described calculated f:m ratios, as well as the percentage of women in OB/GYN and urology of each career position and reference year, were visualized in R (version 4.4.3) using RStudio (version 2024.12.1) and the packages ggplot2, dplyr, tidyr, and patchwork. Temporal trends across career positions were shown by bar plots generated for each specialty. Numbers greater than 1 indicate a f:m ratio with a larger number of women than men.

### Calculation of BG Indices

The calculated ratios were then used to calculate the previously established academic BG Index [9, 10]. The academic BG Index was used to calculate the gender imbalances of women in academic medicine by taking the lower career positions into account:

$${\text{Academic BG Index}} = \frac{{\frac{{{\mathrm{f}}:{\text{m ratio chairs}}_{{{\text{field of medicine}}}} }}{{{\mathrm{f}}:{\text{m ratio specialist physicians}}_{{{\text{field of medicine}}}} }}}}{{{\mathrm{f}}:{\text{m ratio medical students}}_{{{\mathrm{country}}}} }}$$

In this study, the BG Index was modified. In comparison to the academic BG Index, the BGclin Index shows the gender imbalances of leadership positions in clinical medicine, as the numerator is comprised of the f:m ratio of physicians in leadership positions of their respective specialty in general hospitals, as opposed to physicians holding the position of the chair of their respective specialty in a university hospital. It is used to calculate the gender imbalances of women working in clinical medicine by taking leading career positions besides university chair positions into account. To calculate the BGclin Index, the f:m ratio of physicians in leadership positions is divided by the f:m ratio of specialized physicians. The quotient is then divided by the f:m ratio of medical students. The BGclin Index was calculated for the specialties of gynecology and urology for the year 2024 as follows:

$${\text{Clinical BG Index}} = \frac{{\frac{{{\mathrm{f}}:{\text{mratio leadership positions}}_{{{\text{field of medicine}}}} }}{{{\mathrm{f}}:{\text{m ratio specialist physicians}}_{{{\text{field of medicine}}}} }}}}{{{\mathrm{f}}:{\text{m ratio medical students}}_{{{\mathrm{country}}}} }}$$

A BG Index of 1 represents gender parity in higher positions with lower positions taken into account, while a BG Index of < 1 indicates a greater amount of male physicians in higher positions, and a BG Index of > 1 represents a greater amount of female physicians in top positions. Subsequently, the indices were compared, assessing both the vertical development (change over years) and a horizontal state (differences between specialties). The academic BG and BGclin Index were visualized graphically in R (version 4.4.3) using RStudio (version 2024.12.1) and the packages ggplot2, dplyr, tidyr, viridis, and grid.

### Descriptive spatio-temporal analysis of gender differences

Additionally, we determined the proportion of female department chairs in OB/GYN and urology for each university and each evaluated year between 2004 and 2024, to examine descriptive spatial patterns in relation to temporal trends across Germany. In most institutions, the chair position is held by a single individual; therefore, if a woman occupies this role, the percentage is recorded as 100%. At universities with multiple, or jointly appointed chairs, the percentage reflects the share of women among all chair holders, yielding lower values (e.g., 25% when one out of four chair positions is held by a woman).

These values were plotted on maps to visualize both geographic variation and changes over time in female representation at the department-chair level. University-level data were further summarized at the federal-state level, and an additional combined map was created to illustrate overlaps and differences between the two specialties throughout the study period.

All analyses and visualizations were conducted in R (version 4.4.3) using RStudio (version 2024.12.1) and the packages readr, dplyr, tidyr, ggplot2, viridis, sf, rnaturalearth, rnaturalearthdata, and rgeoboundaries. Geospatial data were sourced from publicly available repositories provided by the rnaturalearth and rgeoboundaries databases, and all maps were produced using consistent geographic boundaries and coordinate systems to ensure comparability across years.

### Statistical analysis of gender differences in chair positions

Statistical analysis of chairs was performed with IBM SPSS Statistics v32. Cochran’s Q test was used to perform complete case analysis and Chi-squared test was used for robustness analysis. For pairwise comparison, only continuous occupancy of the chair was considered. The following chairs were thus not taken into account: urology: University of Augsburg: 2019, female; 2024, female. University of Bielefeld: 2024, male. University of Marburg: 2004, male; 2014, male; 2024, male. OB/GYN: University of Augsburg: 2019, male; 2024, male. University of Bielefeld: 2024, female; University of Bonn: 2019, male; 2024, male. University of Oldenburg: 2019, female; 2024, female. Significance values have additionally been adjusted by the Bonferroni correction for multiple tests.

## Results

### Gender distribution at higher career levels favors men

The number of medical students across Germany from 2004 to 2024 increased from 79,866 to 117,342. Since 2004 the number of female students increased from 46,939 to 76,046, leading to a shift of the f:m ratio from 1.426 to 1.842 (Table 1).

**Table 1:** Female to male ratio (f:m) of medical students, specialized physicians, and chair holders in the specialties of obstetrics (OB), gynecology (GYN), and urology

Year	Specialty	Medical students			Specialized physicians			Physicians in a leadership position			Chair holders		
		f	m	f:m	f	m	f:m	f	m	f:m	f	m	f:m
2004	OB/GYN	46,939	32927	1.426	2097	2477	0.847	103	791	0.130	2	37	0.054
	Urology				215	1654	0.130	6	378	0.016	0	35	0.000
2009	OB/GYN	49022	30907	1.586	2723	2174	1.253	93	652	0.143	2	38	0.052
	Urology				305	1724	0.177	9	329	0.027	1	33	0.030
2014	OB/GYN	53352	34,511	1.546	3513	2129	1.650	165	657	0.251	4	34	0.118
	Urology				467	1899	0.246	13	375	0.035	1	34	0.029
2019	OB/GYN	61700	37036	1.666	4182	2034	2.056	189	627	0.301	9	33	0.273
	Urology				640	2047	0.313	18	397	0.045	2	35	0.057
2024	OB/GYN	76.046	41296	1.841	4596	1925	2.388	251	559	0.449	13	30	0.433
	Urology				764	2116	0.361	29	381	0.076	2	36	0.056

In 2004, there were 4547 OB/GYN specialists working in a stationary field in German hospitals in total, with an increase to 6521 by 2024. The number of female specialists increased from 2097 in 2004 to 4596 in 2024, with a change of the f:m ratio from 0.847 to 2.388. When focusing on the physicians in leadership positions in the same specialty, the number of total physicians in leadership positions decreased from 894 to 810 in the years of 2004 to 2024. Still, the amount of female physicians in leadership roles increased from 103 to 251, which equates to a f:m ratio of 0.449 in 2024, compared to 0.13 in 2004. The number of chair holders from 2004 to 2024 increased from 39 to 43. Interestingly, the amount of female chair holders rose drastically with an increase from 2 female chair holders in 2004 to 13 female chair holders in 2024, leading to an increase of the f:m ratio from 0.054 to 0.433.

**Fig. 1:**
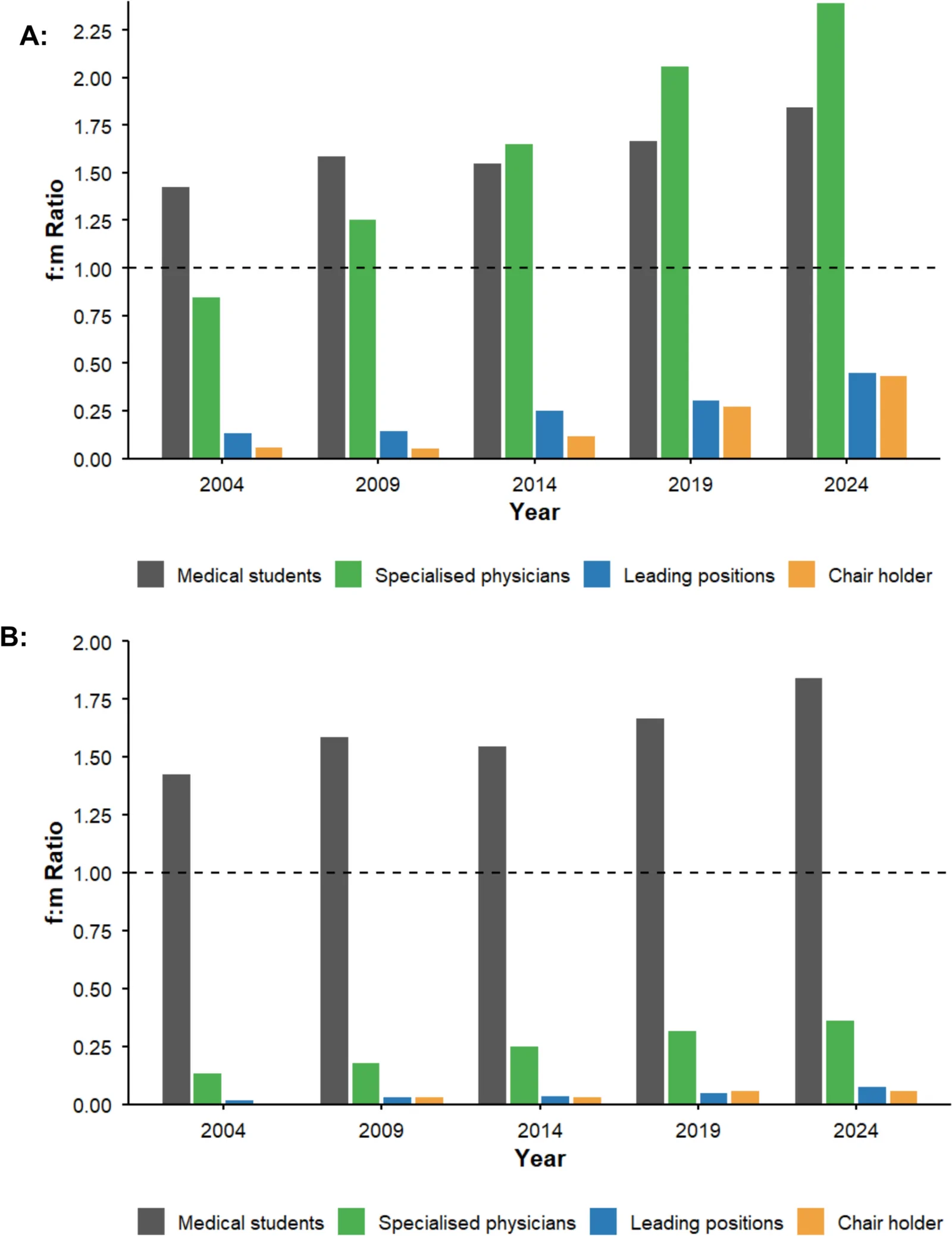
Gender representation in obstetrics (OB) and gynecology (GYN) (**A**), and urology (**B**) by career level (2004–2024). Gender parity would be represented by a female-to-male (f:m) ratio of 1

The number of specialized urologists across Germany from 2004 to 2024 increased from 1869 to 2880. Since 2004, the number of female specialists rose from 215 to 764, leading to an increase of the f:m ratio from 0.13 to 0.361. In 2004, there were 384 physicians in leadership positions total, compared to 410 in 2024. Only 8 of the physicians in leadership positions were female in 2004. The number rose slightly, and by 2024, there were 29 female physician leaders, expressing an increase of the f:m ratio from 0.016 to 0.076. When focusing on the chair holders, all 35 chair holders were male in 2004. The number of chair holders, including the number of female chair holders, rose to 38 chair holders by 2024 in total, 2 of which being female. Therefore, the f:m ratio rose from 0 to 0.056 in 2024 (Fig. 1).

### Changes in the distribution of f:m chair holders toward equality in OB/GYN

Statistical analysis of gender differences in chair positions of OB/GYN and urology (Table 2) revealed that the null hypothesis (categories of the respective year occur with equal probabilities) was rejected in every year (*p* < 0.001), thus confirming that the residual is statistically significant. However, the year 2024 in OB/GYN shows a slight trend toward equality (*p* = 0.005).

**Table 2:** Chi-square test statistics of obstetrics (OB), gynecology (GYN) and urology. df (degrees of freedom), Asymp. Sig. (asymptotic significance/p-value)

	2004	2009	2014	2019	2024
OB/GYN					
Chi-square	29.432^a^	29.432^a^	22.730^a^	14.297^a^	7.811^a^
df	1	1	1	1	1
Asymp. Sig.	< .001	< .001	< .001	< .001	.005
Urology					
Chi-square	^b^	30.118^c^	30.118^c^	30.118^c^	30.118^c^
df		1	1	1	1
Asymp. Sig		< .001	< .001	< .001	< .001

^a^0 cells (0.0%) have expected frequencies less than 5. The minimum expected cell frequency is 18.5

^a^^b^Variable is constant. Chi-square test cannot be performed ^c^0 cells (0,0%) have expected frequencies less than 5. The minimum expected cell frequency is 17.0

Complete case analysis with Cochran’s Q test revealed significant differences between OB/GYN and urology (Table 3). The null hypothesis (the distributions of 2004, 2009, 2014, 2019 and 2024 are the same for the specified categories) was rejected for OB/GYN (*p* < 0.001), while it was retained for urology (*p* = 0.406).

**Table 3:** Related-samples Cochran’s Q test summary of obstetrics (OB), gynecology (GYN) and urology. Asymp. Sig. (asymptotic significance/p-value)

	OB/GYN	Urology
Total N	37	34
Test statistic	22.857	4.000
Degree of freedom	4	4
Asymp. Sig. (2-sided test)	< .001	.406

Pairwise comparison showed that significant changes in distribution of male and female chair holders occurred between 2004 and 2024 (*p* < 0.001), 2009 and 2024 (*p* < 0.001), and 2014 and 2024 (*p* = 0.003) for OB/GYN (Table 4). For urology, the null hypothesis was retained for every pairwise comparison (Table 5).

**Table 4:** Cochran’s Q pairwise comparisons of obstetrics (OB), gynecology (GYN

Sample 1–Sample 2	Test statistic	Std. error	Std. test statistic	Sig	Adj. Sig.^a^
2004–2009	.000	.055	.000	1.000	1.000
2004–2014	−.054	.055	−.976	.329	1.000
2004–2019	−.135	.055	−2.440	.015	.147
2004–2024	−.216	.055	−3.904	< .001	.001
2009–2014	−.054	.055	−.976	.329	1.000
2009–2019	−.135	.055	−2.440	.015	.147
2009–2024	−.216	.055	−3.904	< .001	.001
2014–2019	−.081	.055	−1.464	.143	1.000
2014–2024	−.162	.055	−2.928	.003	.034
2019–2024	−.081	.055	−1.464	.143	1.000

*Std* standard, *Sig* significance, *Adj Sig* adjusted significance

*Each row tests the null hypothesis that the Sample 1 and Sample 2 distributions are the same. Asymptotic significances (2-sided tests) are displayed. The significance level is .050. *^*a*^*Significance values have been adjusted by the Bonferroni correction for multiple tests*

**Table 5:** Cochran’s Q pairwise comparisons of urology

Sample 1–Sample 2	Test statistic	Std. error	Std. test statistic	Sig	Adj. Sig.^a^
2004–2009	− .029	.019	–1.581	.114	1.000
2004–2014	−.029	.019	−1.581	.114	1.000
2004–2019	−.029	.019	−1.581	.114	1.000
2004–2024	−.029	.019	−1.581	.114	1.000
2009–2014	.000	.019	.000	1.000	1.000
2009–2019	.000	.019	.000	1.000	1.000
2009–2024	.000	.019	.000	1.000	1.000
2014–2019	.000	.019	.000	1.000	1.000
2014–2024	.000	.019	.000	1.000	1.000
2019–2024	.000	.019	.000	1.000	1.000

*Std* standard, *Sig* significance, *Adj* *sig* (adjusted significance)

*Each row tests the null hypothesis that the Sample 1 and Sample 2 distributions are the same. Asymptotic significances (2-sided tests) are displayed. The significance level is .050. *^*a*^*Significance values have been adjusted by the Bonferroni correction for multiple tests*

**Fig. 2:**
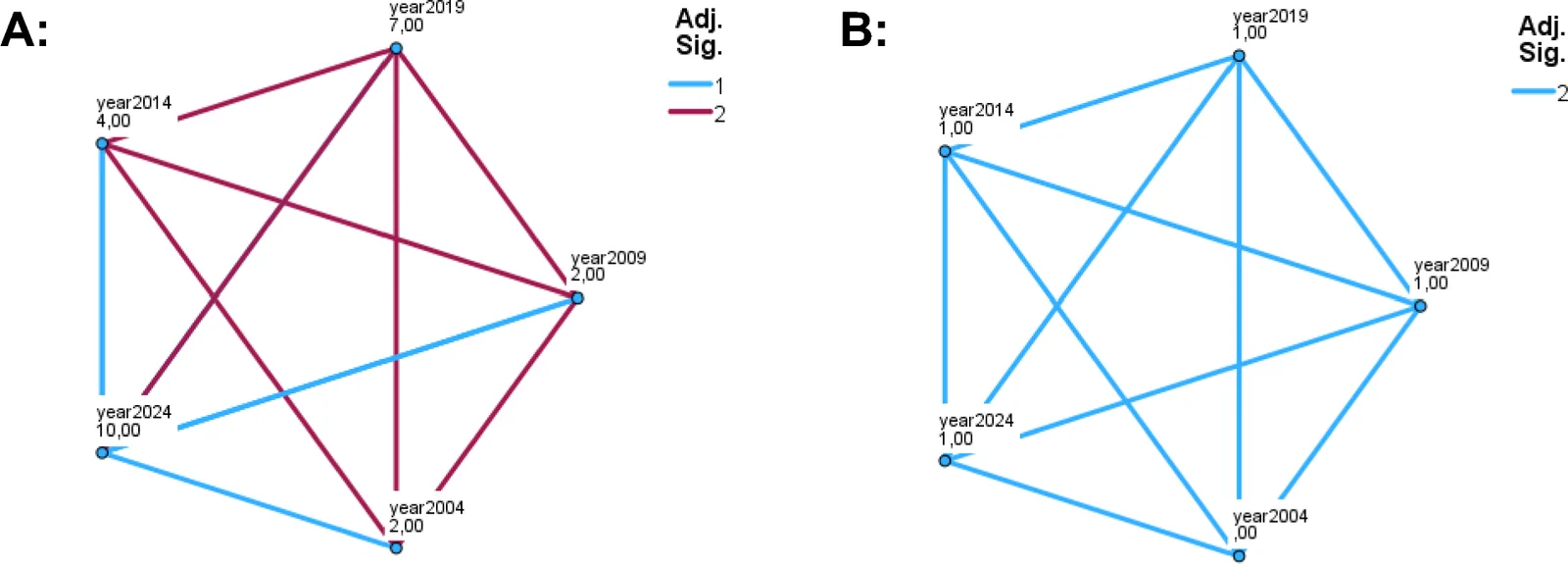
Pairwise comparison of obstetrics (OB) and gynecology (GYN) (**A**), and urology (**B**). Each node shows the sample number of successes. *Adj. Sig* adjusted significance

This is underlined by the number of successes indicated at each node which accounts for up to 1000 successes for OB/GYN, while being limited to 100 for urology (Fig. 2).

### Continuously great gender disparities according to the academic and BGclin Index

**Fig. 3:**
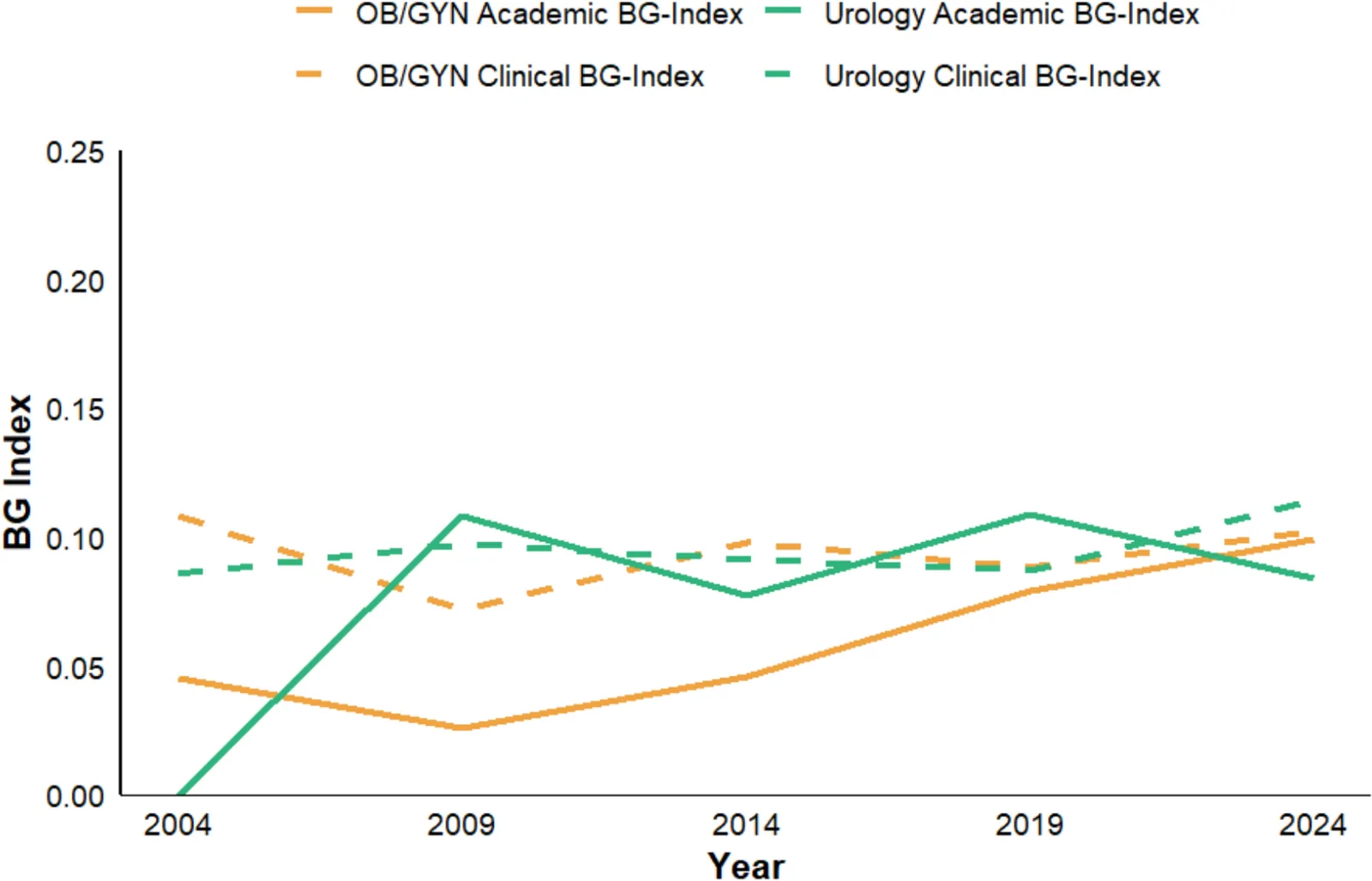
Development of the academic (BG) and clinical (BGclin) Index in obstetrics (OB) and gynecology (GYN), and urology (2004–2024)

In the specialty of OB/GYN, the academic BG Index was 0.045 in 2004, slightly decreasing to 0.026 in 2009, before increasing to 0.099 in 2024. In the specialty of Urology, the academic BG Index of the year 2004 was zero, as there were no female chair holders yet; however, the academic BG Index increased to 0.084 in 2024. It is important to highlight the year of 2009 with an academic BG Index in the specialty of urology of 0.108, as well as 2019 with 0.110, as these are the highest academic BG Indices recorded so far. Compared to the academic BG Index of the specialty of OB/GYN, the academic BG Index of the specialty of urology had a greater increase from 2004 to 2024 (Fig. 3, Table 6).

**Table 6:** Academic (BG) and clinical (BGclin) Indices in obstetrics (OB), gynecology (GYN) and urology 2004–2024

	OB/GYN		Urology	
Year	BG Index	BGclin Index	BG Index	BGclin Index
2004	0.045	0.108	0	0.086
2009	0.026	0.072	0.108	0.097
2014	0.046	0.098	0.077	0.091
2019	0.080	0.088	0.110	0.087
2024	0.099	0.102	0.084	0.114

In OB/GYN, the BGclin Index declined slightly from 0.108 in 2004 to 0.102 in 2024, whereas in urology, it increased from 0.086 to 0.114 over the same period. Both specialties show similar BGclin Indices over the years; however, the BGclin Indices of urology show a greater increase and hold the greatest index determined so far of 0.114 in 2024.

Compared to the academic BG Index, the BGclin Index was greater in both specialties in 2004 and 2024, which highlights the greater gender disparities in higher academic positions. In the specialty of OB/GYN, the BGclin Index was consistently higher than the academic BG Index over the years.

The most recent academic BG Index values (2024), 0.099 in OB/GYN and 0.084 in urology, as well as the corresponding BGclin Index of 0.102 and 0.114, indicate a persistent and substantial gender disparity in both specialties.

### Absence of a descriptive spatial pattern in chair holder positions across Germany

**Fig. 4:**
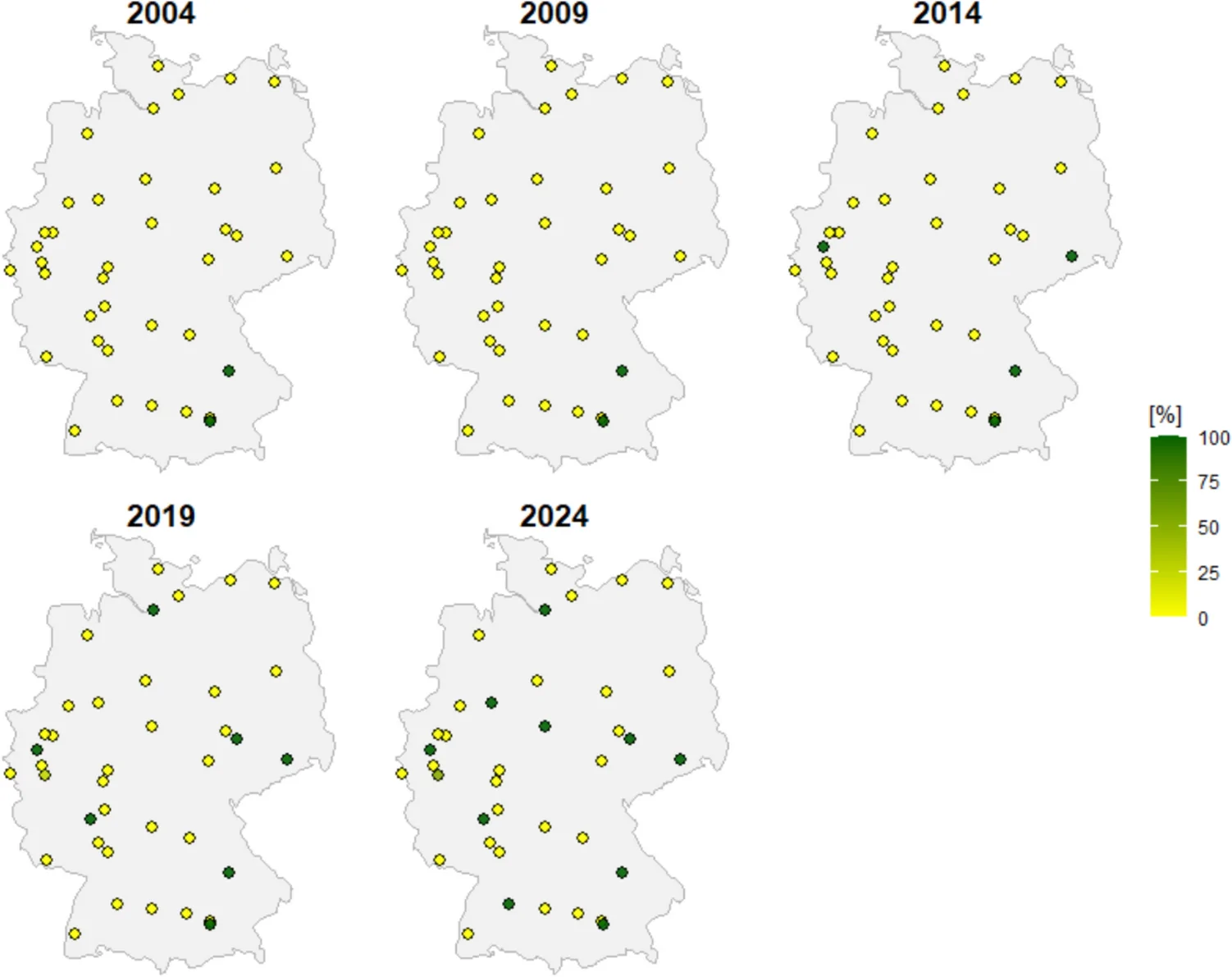
Illustrative spatial analysis of medical faculties across Germany of the percentage of female chair holders in the department of obstetrics (OB) and gynecology (GYN) (2004–2024). In case of shared positions or multiple chair holders, the percentage of female chair holders (0–100%) is calculated

By determining the proportion of female department chairs in OB/GYN and urology for each university and each evaluated year between 2004 and 2024, we examined descriptive spatial patterns in relation to temporal trends across Germany. Furthermore, a descriptive spatial analysis revealed an absence of spatial patterns in German chair holder positions. The descriptive spatial–temporal analysis of the specialty of OB/GYN shows a drastic increase of female chair holders of 550% (from 2 to 13 female chair holders) over the years. When examining individual university locations across Germany, no descriptive spatial trend in chair holder positions could be identified. Although most female chair holders were appointed in the former West German states, there is no systematic West–East divide, since Saxony has had female chair holders since 2014. This indicates a random spatial distribution rather than a consistent regional pattern (Fig. 4).

**Fig. 5:**
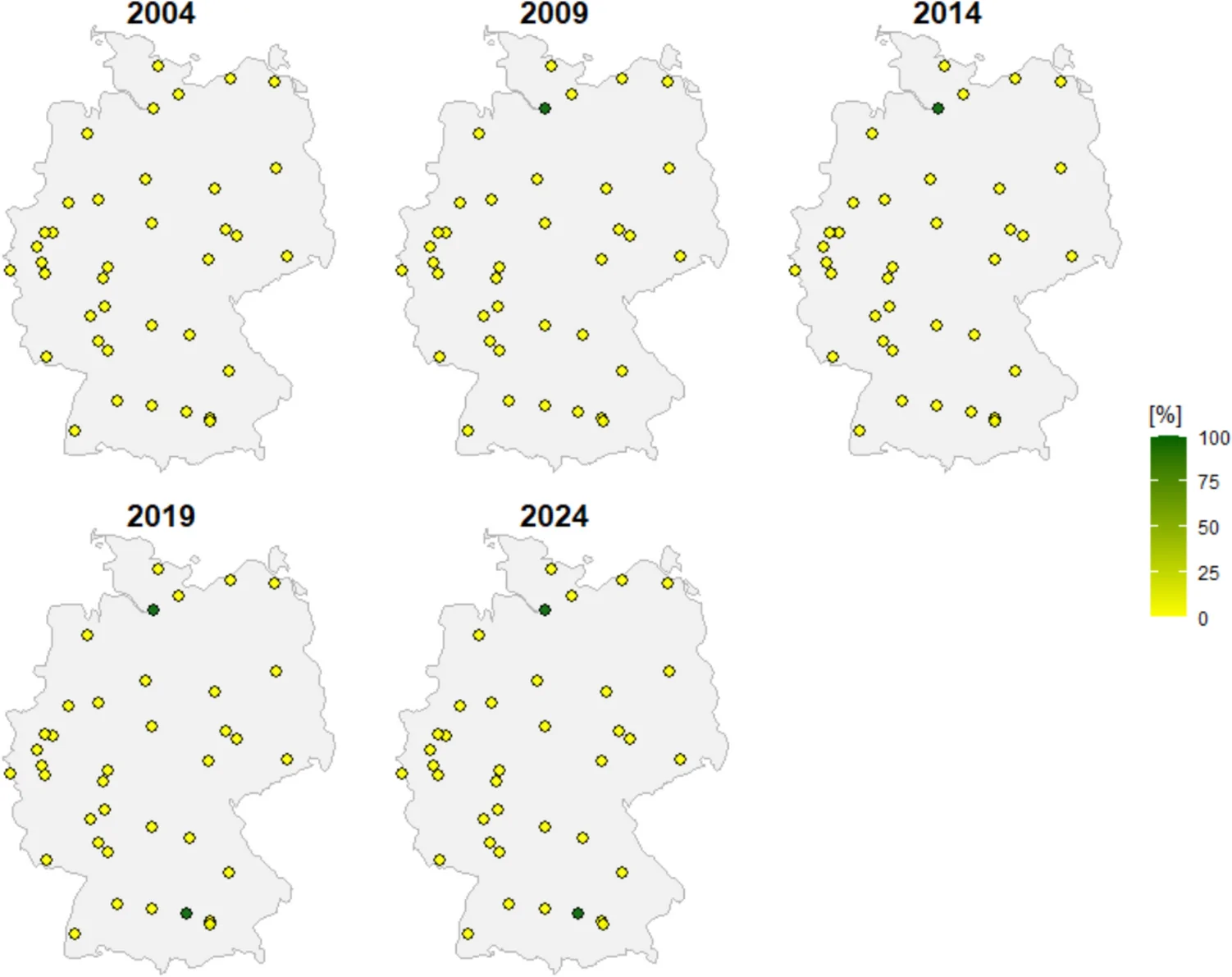
Illustrative spatial analysis of medical faculties across Germany of the number of female chair holders in the department of urology (2004–2024). In case of shared positions or multiple chair holders, the percentage of female chair holders (0–100%) is calculated

Over time, the number of female chair holders in the specialty of urology increased only marginally, from none in 2004 to two by 2024. Both appointments occurred in the former West German states, first in Hamburg (2008) and later in Bavaria (2019) [14]. Given the very small number of cases and their confinement to individual locations, this distribution does not indicate a descriptive spatial pattern or a systematic West–East divide (Fig. 5).

Taken together, these data show a notable difference between the specialties of OB/GYN and urology, with a greater increase of the number of female chair holders in the specialty of OB/GYN. Of note, OB/GYN had already appointed more female chair holders at the first analyzed year (Figs. 4, 5).

**Fig. 6:**
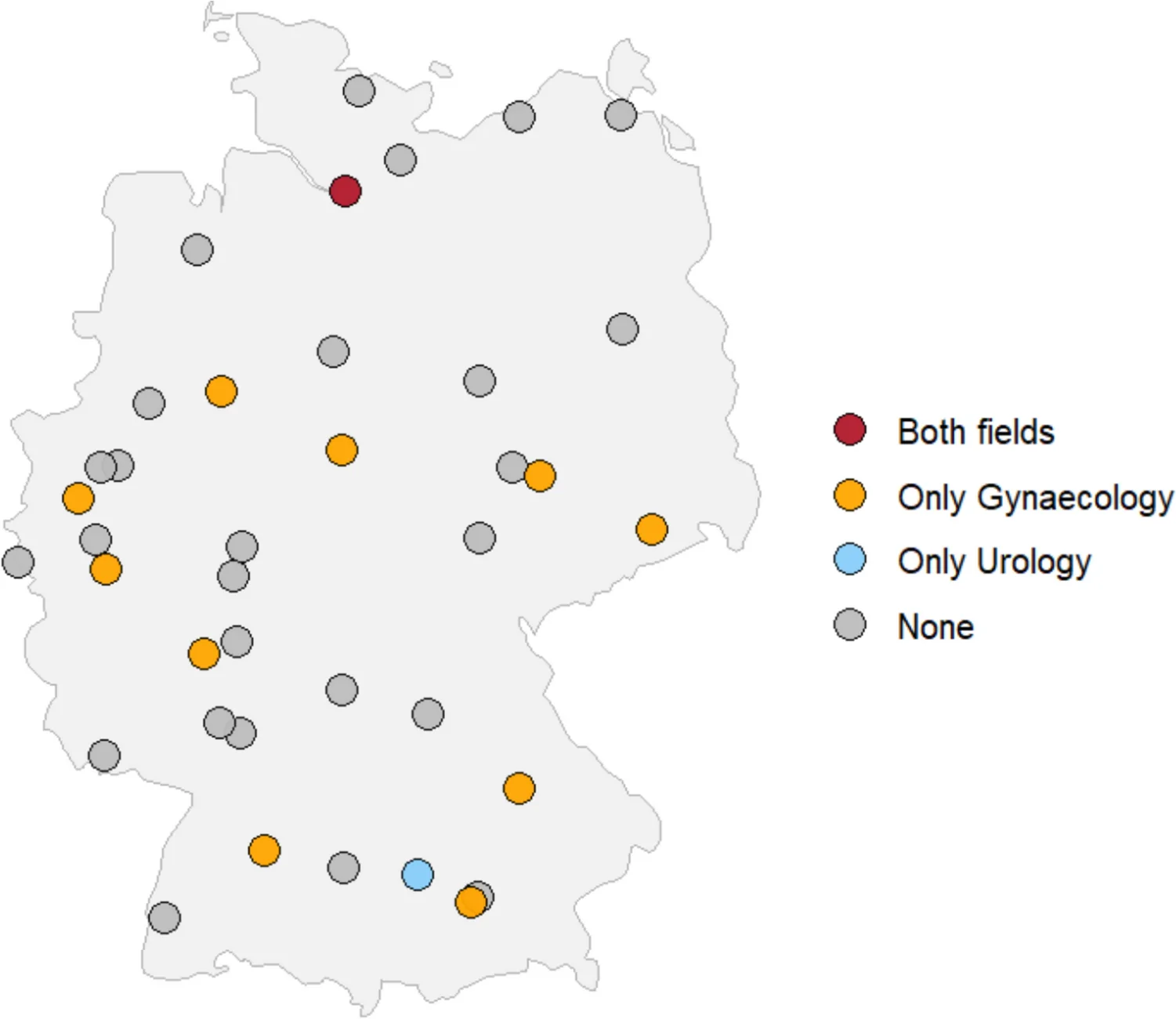
Illustrative spatial distribution of female chair holders at 38 medical universities across Germany in the departments of Obstetrics (OB), Gynecology (GYN) and Urology in 2024

There is only one university in Germany, the University of Hamburg, which appointed female chair holders in both specialties, OB/GYN and urology. Since 2004, ten additional universities appointed female chair holders in OB/GYN, whereas only one additional female chair holder was appointed in urology. However, the majority of German universities had no female chair holders in either specialty over the examined time period (Fig. 6).

## Discussion

Throughout the years, the number of female medical students has risen to the point that female medical students outnumber their fellow male students by 64%. Even though the number of female specialized physicians has increased, there is little progression of the gender parity in leadership positions and chair holders. Both the academic and the BGclin Index show great gender disparities in the fields of OB/GYN and urology. In some cases, the BG Index has even decreased since the first calculation in 2004 (BGclin Index of OB/GYN). The fact that the BG indices in urology show a greater increase since 2004 compared to the BG indices of OB/GYN can be attributed to both the nonexistence of female chair holders in 2004 in urology, and the great number of specialized female physicians in the field of OB/GYN, resulting in a greater denominator in the equation and thus a lower BG Index, since the quotient of a division decreases with the increase of the denominator.

Additionally, there is no notable difference of gender parity in academic positions within Germany, as no clear descriptive spatial trend of female chair holder positions can be found for both medical fields.

### BG Indices and f:m ratio exhibit temporal stagnation

Since 2004, the number of female students increased drastically, leading to a shift of the f:m ratio from 1.426 to 1.842. From 2004 to 2024, the f:m ratios increased among hospital specialists (OB/GYN: 0.847 to 2.388, urology: 0.13 to 0.361), leadership positions (OB/GYN: 0.13 to 0.449, urology: 0.016 to 0.076) and chair holders (OB/GYN: 0.054 to 0.433, urology: 0 to 0.056). However, women outnumber men only at the student and specialist level in the field of OB/GYN, while they are still underrepresented as specialists in urology and higher job positions. Interestingly, the gender distribution at higher career levels not only favors men but is significantly different between the specialties of OB/GYN and urology. While significant changes in the distribution of male and female chair holders could be observed in OB/GYN, these changes were not detectable for urology. Reasons for these differences will be discussed below. Similar to the f:m ratio, the BG Indices show fewer representation of female physicians as the career position advances. Even though OB/GYN is the department with the highest percentage of female practitioners (with the exception of the department of child and adolescent psychiatry which has a f:m ratio of 2.66 in 2024, while OB/GYN has a f:m ratio of 2.388), it does not have the highest academic BG Index compared to other departments [4]. For most of the last 2 decades, the academic BG Index of the department of OB/GYN is lower than the academic BG Index of the field of urology (Fig. 2). This can be explained by the fact that the f:m ratio of specialized physicians is much higher in the field of OB/GYN than in the field of urology. Since the f:m ratio of the specialized physicians is one of the denominators of the fraction, a bigger denominator equates to a smaller result. Gender parity in the department of OB/GYN would be reached with 35 female academic chair holders out of 43 total chair holders instead of 13, whereas for the department of urology in the same year, gender parity would be achieved with 16 female academic chair holders out of 38 total chair holders instead of 3. This shows the difference between only taking the number of chair holders into account and dividing it into two equal parts, vs. additionally taking the number of female specialized physicians and medical students into account, hence determining the parity with consideration of the gender composition of lower career positions. Since the last calculation of the academic BG Index of OB/GYN in 2013 [10], the number of female chair holders in the OB/GYN increased by 9 (4 in 2013, 13 in 2024). Interestingly, the academic BG Index has only risen slightly in both specialties. This can be explained by the greater increase of female students, as well as the increase of female specialized physicians, which increases the denominator of the equation. The academic BG Index analyzes only the highest career position, thus could be lagging behind the increased number of female medical students, and career progression dynamics. Thus, the BGclin Index was introduced to analyze leadership positions that can be reached more rapidly. When addressing the BGclin Index, taking the gender disparities of leadership positions of clinical medicine into account, it is apparent that the gender equilibrium is far from being reached (Fig. 3, Table 6).

When comparing the f:m ratio of specialized OB/GYN physicians in Germany with that in the United States, it becomes apparent that a similar increase occurred in the United States, rising from 15% in 1973 to 67.3% in 2022 over the past 50 years [15]. Based on current trends, fewer men are applying for OB/GYN residency due to factors such as low income, reduced prestige, and limited career prospects. As a result, male residents are projected to comprise only 1% of the field within 10 years, falling well below the critical mass of 15% needed to sustain participation and engagement [15, 16]. Interestingly, in the field of urology, a rise in residents can be found in Spain (67% female residents by 2022), and Belgium (46% female residents by 2022), while the number of specialized physicians has only increased slightly [17]. Contrary to the European countries, the number of female practicing urologists in the United States has increased, whereas the increase of female residents and applicants have slowed from 2007 to 2020 [18]. When comparing these results to the previously published academic BG Indices of OB/GYN, it becomes apparent that since the first calculated academic BG Index in 1998 (0), the index has risen slightly (0.099 in 2024) [10]. The same applies to the field of urology, which likewise started with an academic BG Index of 0 in 2000, that rose to 0.084 by 2024 [9]. It is important to note that urology shows the biggest academic BG Index ever recorded in 2019 with 0.110. When evaluating these results, the slow progression of gender parity becomes apparent, as the academic BG Index has only risen slightly from the point it was first calculated in 1998. Continuing in this pace, it is estimated that gender parity would not be reached until 2051 [19].

### No spatial differences of gender parity in top positions in Germany

For the first time, this study investigates descriptive spatial differences in gender parity in the fields of OB/GYN and urology across Germany. In both fields, no descriptive spatial differences between North and South Germany or old and new federal states became apparent. Even though a study from 2024 shows that 16 out of 19 of the universities with an above-average number of female leaders are located in the old federal states, indicating a distinction between old and new federal states, this cannot be replicated in the position of chair holders in the field of OB/GYN and urology [6, 13]. When discussing spatial patterns outside of Germany in the United States, a notable difference can be found between rural and dense urban areas, with most female physicians practicing in dense urban areas, which can be acquitted to a variety of reasons, including association with an academic practice or center, or job opportunities for a partner [20]. The advantage of the descriptive spatial analysis is that it indicates general societal gender inequalities rather than historically driven spatial patterns across Germany. Moreover, it highlights that in some federal states (e.g., OB/GYN: Schleswig-Holstein, Mecklenburg-Vorpommern, Bremen, Lower Saxony, Berlin, Brandenburg, Saxony-Anhalt, Thuringia, and Saarland; urology: Schleswig-Holstein, Mecklenburg-Vorpommern, Bremen, Lower Saxony, Berlin, Brandenburg, Saxony-Anhalt, Thuringia, Hessen, North Rhine-Westphalia, Rhineland-Palatinate, Baden-Württemberg and Saarland), no woman has ever held a chair position.

### Academic drivers of gender imbalance at higher career levels

Various factors, among them differences in the experience during internships, higher interest rates in the respective fields, differing pre-residency concerns, differences in gender-based discrimination, as well as seeing primarily female or male patients, respectively, contribute to a preference of female students to pursue a residency and career in OB/GYN and of male students in urology, respectively [21–29]. Three main academic factors drive or reinforce the gender imbalance at higher career levels, such as chair positions, alongside family-related considerations influencing the career decisions of women. These factors include 1) male-dominated decision-making groups within appointment committees and non-representative speaker panels [30, 31], 2) imbalances in qualifications and publication records resulting from career-family dynamics [32], and 3) the persistent gender pay gap [33, 34].

As described, the gender composition of appointment committees remains a critical structural factor for women’s advancement to senior academic positions [7]. The pool of qualified female candidates suitable for high academic positions is large: in 2022, on average 37% of all female senior physicians were either habilitated or holding professorships [31]. With most appointment committees consisting of predominantly male members, it can be especially daunting for women to appear in front of such a group, affecting the outcome of the appointment process [31]. The decision-making group in an appointment committee consists usually of professors of the university. A study done in 2024 by the German association of female doctors revealed that only three faculties had gender parity in their appointment committees [6, 7, 31]. When questioned, some faculties stated the reason for the gender disparity was too few female professors, with the ones that were present often declining the positions in appointment committees due to the additional workload which accrues to the heavy clinical workload, unpaid care work (childcare), and work in patient care and nursing which women tend to take part in more than their male colleagues [6, 35]. Oftentimes, the work in appointment committees was either voluntary or with hourly exemption from the work as a physician [31]. Only one out of 15 physicians was granted a full-time leave of absence to pursue the work as an equal opportunity commissioner [31]. The responsibilities and rights of the equal opportunity commissioner also vary with the different institutions: while a consultative role is often given, the right to vote or veto in appointment committees is only given in roughly a third of the faculties [36]. It is argued that men choose in favor of other men because of exclusive network practices by searching for candidates in their own circle, sexist perceptions held by both men and women alike, which view the level of ambition of female candidates as lesser than their male counterparts, and view women as having less authority as head of the department [37, 38].

It is crucial to not only mention the parties responsible for deciding who will be appointed to a higher position, but also the different qualifications that are looked for in candidates. Both clinical experience and research are important for applicants. However, publications show gender biases starting at a lower acceptance rate for authors self-reporting as women (46% acceptance rate for female authors vs 55% for male authors) [32]. Publishing profoundly impacts career advancement and is essential for promotion as it is a well-established metric for productivity in academic medicine [39]. A study from 2022 revealed not only that men publish more than women, but the fact that men with children publish significantly more than women with children, which might be the result of women taking on more of the childcare and domestic duties [40]. Even though professional childcare is a desire of most female OB/GYN physicians, the majority of institutions or employers do not offer it [41]. A promising solution to the difference of productivity of men vs. women after childbirth is dependent-care policies to help parents, especially mothers, to better meet their professional demands [42]. To lessen the impact of pregnancy and childcare on the H-impact (an academic impact on productivity, and citation impact in research), alternative monitoring for academic promotion should be offered, for example through the “clinical educator track”, which clearly states the impact of parental leave [43]. Furthermore, studies in urology and gynecologic oncology show increasing female authorship as first authors, yet women remain underrepresented as last authors [39, 44]. To address the issue of the inequitable publication process, journals like Nature are actively working to diversify their pool of reviewers and minimize biases through unconscious-bias training [32].

Another well-known disadvantage for women in the workplace is the gender pay gap. Although the gender pay gap in academic medicine is lower than the average gender pay gap in the United States, (90 cents on the dollar vs. the average of 82 cents on the dollar) [33], in the field of urology, the median salary difference still adds up to be $81,578 per year, even after taking salary determining factors like academic rank, specialty and work duties (clinical, research, administrative and education) into account [33, 34]. Equal opportunities for men and women promote entrepreneurship and innovation, economic development and benefits, and population health, which is why women should not have to work harder than men to reach higher job positions, while also earning less than their male colleagues [45–47]. The financial losses for women are not the only consequence of the gender pay gap, since women self-report a lower career satisfaction and are less likely to stay in academic medicine than their male counterparts, which is partly caused by higher rates of burnout due to a higher workload overall, as women tend to take on more unpaid care work, adding up to an average of 8.5 more hours of household tasks than men per week [34, 35, 48]. To combat the gender pay gap, academic institutions should standardize the initial salaries and address existing inequities [49].

### Emerging work-time models gaining importance

The reasons for the leaky pipeline (the phenomenon in which many women “drop out” of the path to academic job promotion, leading to an underrepresentation at the top levels) [50] in academic medicine is not solely due to the fact that it is harder for women to climb the academic ladder [51]. Often, the ambitions of women in medicine are lower than the ones of their male colleagues [52]. Women plan to have a family earlier in their career, while men focus on career promotion during the same time period [50, 52]. Career advancement is only desirable for 47% of all questioned physicians, while the acquisition of an academic title is still attractive for 65% [52]. This proves that the balance between working and raising a family is especially important for women, and with that, flexible and reliable working hours are of utmost importance to achieve gender parity in academic medicine [53]. A realistic solution to this problem is the model of top-sharing, in which a labor-intensive position is split between two or more people, sharing the responsibilities and organizational tasks [6]. There are many different models as to how it might manifest, with two people working full time as one popular model, or with both parties working part-time [54]. For colleagues, patients, and the hospitals, this model brings many positive aspects: two people sharing a top position means knowledge and experience is doubled, the availability increases and decision-making is accelerated [54, 55]. It may also expand the continuing education authorization that is needed to further educate resident physicians [54]. In a survey done by the “German female physician association”, 84.9% of all questioned physicians (both female and male) would apply for a shared top position [56]. It shows that many women are willing to pursue top positions when sharing is an option, but traditional models and lack of institutional support often deter them [56]. Top-sharing directly addresses structural barriers that disproportionately affect women, such as a lack of supportive models for combining leadership with caregiving, traditional hierarchies and unrealistic time expectations [55].

The first study on the academic BG Index in the field of OB/GYN addresses the succession and need of mentoring programs, naming the Rahel Hirsch scholarship of the medical school of the Charité as a positive example [10]. With the updated BG Index and considering that 35% of female medical students report having worried about the impact their sex would have on their career, along with female OB/GYN specialists having lower confidence in surgical and practical skills than their male colleagues, it is apparent that the need of mentoring programs is a prevailing topic [26, 57]. Participants of mentorship programs like the FLEX Leadership Development Program (encouraging women to build leadership skills) report improved situational awareness, communication skills and self-confidence, which helped them better advocate for themselves [58]. It is important to note the difference between mentoring and sponsoring programs, as both are important and helpful for women to advance to higher positions in their academic career [8]. While mentoring programs are designed to advance the personal growth by coaching the mentee by giving feedback and advice, sponsorships involve people in higher (academic) positions by advocating for the person in the lower position in key roles and grand opportunities [8]. Both mentorship and sponsorship should be provided by institutions to promote gender equity, ideally providing women access to influential people through the mentorship [58, 59]. It is important to start these programs as early as possible, as some gender biases targeting women commence with the beginning of medical school [50]. Statistical analysis revealed that participants of national career development programs were more likely to be promoted to Associate Professor than non-participant women and men, and within 10 years as likely as men and more likely than non-participant women to be promoted to Full Professor [60]. While participating women had a significantly higher chance to be promoted into higher academic positions than non-participating women, the difference between men and participating women is not as eminent, which indicates that national career development programs are suitable to help level the field for career advancement, and broaden diversity in leadership positions within academic medicine [60].

### Limitations and outlook

Several study limitations must be acknowledged. First, the dataset for the spatio-temporal analysis comprises only five measurement points, assessed at 5-year intervals over a 20-year period. Given this limited number of observations, advanced time series analyses such as ARIMA model fitting, the Augmented Dickey–Fuller stationarity test, or autocorrelation function (ACF/PACF), and even linear trend regression analyses are not statistically appropriate, as these methods require a minimum of at least 10 to 30 data points to yield reliable estimates. Thus, our analyses are partially confined to descriptive statistics. Future studies should address this issue by incorporating more measure points for in-depth statistical analysis. Second, this study is limited by the fact that only two genders are provided by the German Medical Association. We recognize that this does not cover all gender identities, which should be addressed in future studies. Third, OB/GYN and urology were chosen as the two comparator specialties based on previous studies [10]; however, other predominantly male specialties were not included in this study. This limits the generalizability of this study and future studies should compare the specialty of OB/GYN to a broader selection of predominantly male specialties to minimize a possible selection bias. Fourth, while trends in the United States and Europe are outlined in the discussion, this study only analyzes Germany. Future studies could apply the indices applied in this study to analyze European-wide trends and contextualize them in regard to trends in the United States and globally. The indices used in this study are a helpful tool to assess and document changes in the upcoming years. While long-term goals, such as the elimination of the gender pay gap in academic medicine, diversification of reviewer pools, as well as parity in chair positions and other leadership roles should be pursued, e.g., by implementing targeted career development programs, the success of these implementations needs constant monitoring to evaluate their effectiveness. The standardized implementation of the methods used in this study, e.g., every 5 years and spanning all specialties, would provide comprehensive analysis to either validate the success of implemented measures or express the need to further advance female career progression.

## Conclusion

For over 25 years, female medical students have outnumbered their male fellow students. While this trend can be seen in the number of f:m specialized physicians, female physicians are still underrepresented in top positions. However, in contrast to urology, a significant change in the temporal distribution of male and female chair holders toward equality could be observed in OB/GYN. A preference of female students to pursue a residency and career in OB/GYN and of male students in urology is contributed to different factors, such as higher interest rates and differences in gender-based discrimination [21–29]. By using the academic and BGclin Index in the fields of OB/GYN and urology, this study shows the continuous gender inequity and stagnation of progression in academic and clinical medicine. Moreover, spatial analysis showed no descriptive spatial difference in progression of gender parity in Germany. After having assessed both BG Indices of the fields of OB/GYN and urology for the last 2 decades and having compared the results to previously determined academic BG Indices of the same fields, it has become apparent that substantial work needs to be done to work toward gender parity. There continues to be a need for further research on how to better fight against gender disparity. In addition to that, a concerted effort must be made to address the many known factors in all stages of the career withholding women from rising to higher academic positions to provide full inclusion to all physicians in modern healthcare. Proven solutions start as early as medical school through mentoring programs, continue with dependent-care policies to facilitate career progression for academic parents, and the combat of the gender pay gap. Finally, it is important to strive toward gender parity in appointment-commissions and offer work-time models such as top-sharing positions to actively promote gender parity in top positions of academic and clinical medicine.

## Supplementary Information

Below is the link to the electronic supplementary material.

Supplementary material 1 (DOCX 52 KB)

Supplementary material 2 (DOCX 98.2 kb)

## Data Availability

Number of medical students was accessed on the DeSTATIS database, by the Federal Ministry of Internal Affairs. URL: https://genesis.destatis.de/datenbank/online/statistic/21311/table/21311-0012#filter=JTdCJTIyaGlkZUVtcHR5Q29scyUyMiUzQWZhbHNlJTJDJTIyaGlkZUVtcHR5Um93cyUyMiUzQWZhbHNlJTJDJTIyY2FwdGlvbiUyMiUzQSU1QiU3QiUyMnZhcmlhYmxlSWQlMjIlM0ElMjIyMTMxMSUyM Number and gender of specialized physicians were drawn from the Federal Chamber of Physicians. URL: https://www.bundesaerztekammer.de/fileadmin/user_upload/BAEK/Ueber_uns/Statistik/AErztestatistik_2024.pdf
