# Supplementary material for: Female career advancement in obstetrics/gynecology, and urology: a spatio-temporal comparative study over the last 2 decades in Germany
Source: Arch Gynecol Obstet. 2026 Sep 3;313(1):264. doi: 10.1007/s00404-026-08511-2 (PMC13541887; doi:10.1007/s00404-026-08511-2)
Supplement: Supplementary file 1 [file 404_2026_8511_MOESM1_ESM.docx]

Dataset Figure 1,2

|  | **OB/GYN** | | **Urology** | |
| --- | --- | --- | --- | --- |
|  | **Academic BG-Index** | **Clinical BG-Index** | **Academic BG-Index** | **Clinical BG-Index** |
| **2004** | 0.045 | 0.108 | 0 | 0.086 |
| **2009** | 0.026 | 0.072 | 0.108 | 0.097 |
| **2014** | 0.046 | 0.098 | 0.077 | 0.091 |
| **2019** | 0.080 | 0.088 | 0.110 | 0.087 |
| **2024** | 0.099 | 0.102 | 0.084 | 0.114 |

Dataset Figure 3,5

Gynecology and Obstetrics

| University | State | Lat | Long | % Women 2004 | % Women 2009 | % Women 2014 | % Women 2019 | % Women 2024 |
| --- | --- | --- | --- | --- | --- | --- | --- | --- |
| RWTH Aachen | NRW | 50,77835652 | 6,077349913 | 0 | 0 | 0 | 0 | 0 |
| Universität Augsburg | Bayern | 48,33428338 | 10,8984804 | 0 | 0 | 0 | 0 | 0 |
| Charité – Universitätsmedizin Berlin | Berlin | 52,53348761 | 13,37653383 | 0 | 0 | 0 | 0 | 0 |
| Universität OWL Bielefeld | NRW | 52,03407127 | 8,499472035 | 0 | 0 | 0 | 0 | 100 |
| Ruhr-Universität Bochum | NRW | 51,44549437 | 7,258539248 | 0 | 0 | 0 | 0 | 0 |
| Friedrich-Wilhelms-Universität Bonn | NRW | 50,72696394 | 7,086409882 | 0 | 0 | 0 | 25 | 50 |
| Technische Universität Dresden | Sachsen | 51,02977368 | 13,73152216 | 0 | 0 | 100 | 100 | 100 |
| Heinrich-Heine-Universität Düsseldorf | NRW | 51,19160933 | 6,794132213 | 0 | 0 | 100 | 100 | 100 |
| Friedrich-Alexander-Universität Erlangen-Nürnberg | Bayern | 49,5979124 | 11,00454121 | 0 | 0 | 0 | 0 | 0 |
| Universität Duisburg-Essen | NRW | 51,43410161 | 6,988652213 | 0 | 0 | 0 | 0 | 0 |
| Johann-Wolfgang-Goethe-Universität Frankfurt am Main | Hessen | 50,12723255 | 8,667795684 | 0 | 0 | 0 | 0 | 0 |
| Albert-Ludwigs-Universität Freiburg | Baden-Württemberg | 47,99366233 | 7,845948876 | 0 | 0 | 0 | 0 | 0 |
| Justus-Liebig-Universität Gießen | Hessen | 50,58103967 | 8,677097384 | 0 | 0 | 0 | 0 | 0 |
| Georg-August-Universität Göttingen | Niedersachen | 51,54180152 | 9,937423536 | 0 | 0 | 0 | 0 | 100 |
| Ernst-Moritz-Arndt-Universität Greifswald | Mecklenburg-Vorpommern | 54,09524214 | 13,374584 | 0 | 0 | 0 | 0 | 0 |
| Martin-Luther-Universität Halle-Wittenberg | Sachsen-Anhalt | 51,48621872 | 11,96947785 | 0 | 0 | 0 | 0 | 0 |
| Universität Hamburg | Hamburg | 53,56324742 | 9,988414926 | 0 | 0 | 0 | 100 | 100 |
| Medizinische Hochschule Hannover | Niedersachen | 52,38381904 | 9,804955397 | 0 | 0 | 0 | 0 | 0 |
| Ruprecht-Karls-Universität Heidelberg | Baden-Württemberg | 49,41095315 | 8,706640128 | 0 | 0 | 0 | 0 | 0 |
| Universität des Saarlandes Homburg | Saarland | 49,25531104 | 7,040940668 | 0 | 0 | 0 | 0 | 0 |
| Friedrich-Schiller Universität Jena | Thüringen | 50,92862196 | 11,58169463 | 0 | 0 | 0 | 0 | 0 |
| Christian-Albrechts-Universität Kiel | Schleswig-Holstein | 54,34361082 | 10,11499261 | 0 | 0 | 0 | 0 | 0 |
| Universität zu Köln | NRW | 50,92839842 | 6,929317882 | 0 | 0 | 0 | 0 | 0 |
| Universität Leipzig | Sachsen | 51,33894914 | 12,37867608 | 0 | 0 | 0 | 100 | 100 |
| Universität zu Lübeck | Schleswig-Holstein | 53,83442099 | 10,70369091 | 0 | 0 | 0 | 0 | 0 |
| Otto-von-Guericke-Universität Magdeburg | Sachsen-Anhalt | 52,14035109 | 11,6447011 | 0 | 0 | 0 | 0 | 0 |
| Johannes-Gutenberg-Universität Mainz | Rheinland-Pfalz | 49,99276442 | 8,241587384 | 0 | 0 | 0 | 100 | 100 |
| Medizinische Fakultät Mannheim der Universität Heidelberg | Baden-Württemberg | 49,50085392 | 8,493333347 | 0 | 0 | 0 | 0 | 0 |
| Philipps-Universität Marburg | Hessen | 50,81052134 | 8,773999213 | 0 | 0 | 0 | 0 | 0 |
| Ludwig-Maximilians-Universität München | Bayern | 48,15133363 | 11,57973215 | 0 | 0 | 0 | 0 | 0 |
| Technische Universität München | Bayern | 48,14906522 | 11,5683266 | 100 | 100 | 100 | 100 | 100 |
| Westfälische Wilhelms- Universität Münster | NRW | 51,96344192 | 7,613547468 | 0 | 0 | 0 | 0 | 0 |
| Carl-von-Ossietzky Universität Oldenburg | Niedersachsen | 53,14700353 | 8,183274201 | 0 | 0 | 0 | 0 | 0 |
| Universität Regensburg | Bayern | 48,9969171 | 12,09581898 | 100 | 100 | 100 | 100 | 100 |
| Universität Rostock | Mecklenburg-Vorpommern | 54,08539311 | 12,10114037 | 0 | 0 | 0 | 0 | 0 |
| Eberhard-Karls-Universität Tübingen | Baden-Württemberg | 48,52527152 | 9,057319784 | 0 | 0 | 0 | 0 | 100 |
| Universtität Ulm | Baden-Württemberg | 48,4235755 | 9,95517542 | 0 | 0 | 0 | 0 | 0 |
| Julius-Maximilians-Universität Würzburg | Bayern | 49,78836147 | 9,935281455 | 0 | 0 | 0 | 0 | 0 |

Dataset Figure 4,5

Urology

| University | State | Lat | Long | % Women 2004 | % Women 2009 | % Women 2014 | % Women 2019 | % Women 2024 |
| --- | --- | --- | --- | --- | --- | --- | --- | --- |
| RWTH Aachen | NRW | 50,77835652 | 6,077349913 | 0 | 0 | 0 | 0 | 0 |
| Universität Augsburg | Bayern | 48,33428338 | 10,8984804 | 0 | 0 | 0 | 100 | 100 |
| Charité – Universitätsmedizin Berlin | Berlin | 52,53348761 | 13,37653383 | 0 | 0 | 0 | 0 | 0 |
| Universität OWL Bielefeld | NRW | 52,03407127 | 8,499472035 | 0 | 0 | 0 | 0 | 0 |
| Ruhr-Universität Bochum | NRW | 51,44549437 | 7,258539248 | 0 | 0 | 0 | 0 | 0 |
| Friedrich-Wilhelms-Universität Bonn | NRW | 50,72696394 | 7,086409882 | 0 | 0 | 0 | 0 | 0 |
| Technische Universität Dresden | Sachsen | 51,02977368 | 13,73152216 | 0 | 0 | 0 | 0 | 0 |
| Heinrich-Heine-Universität Düsseldorf | NRW | 51,19160933 | 6,794132213 | 0 | 0 | 0 | 0 | 0 |
| Friedrich-Alexander-Universität Erlangen-Nürnberg | Bayern | 49,5979124 | 11,00454121 | 0 | 0 | 0 | 0 | 0 |
| Universität Duisburg-Essen | NRW | 51,43410161 | 6,988652213 | 0 | 0 | 0 | 0 | 0 |
| Johann-Wolfgang-Goethe-Universität Frankfurt am Main | Hessen | 50,12723255 | 8,667795684 | 0 | 0 | 0 | 0 | 0 |
| Albert-Ludwigs-Universität Freiburg | Baden-Württemberg | 47,99366233 | 7,845948876 | 0 | 0 | 0 | 0 | 0 |
| Justus-Liebig-Universität Gießen | Hessen | 50,58103967 | 8,677097384 | 0 | 0 | 0 | 0 | 0 |
| Georg-August-Universität Göttingen | Niedersachen | 51,54180152 | 9,937423536 | 0 | 0 | 0 | 0 | 0 |
| Ernst-Moritz-Arndt-Universität Greifswald | Mecklenburg-Vorpommern | 54,09524214 | 13,374584 | 0 | 0 | 0 | 0 | 0 |
| Martin-Luther-Universität Halle-Wittenberg | Sachsen-Anhalt | 51,48621872 | 11,96947785 | 0 | 0 | 0 | 0 | 0 |
| Universität Hamburg | Hamburg | 53,56324742 | 9,988414926 | 0 | 100 | 100 | 100 | 100 |
| Medizinische Hochschule Hannover | Niedersachen | 52,38381904 | 9,804955397 | 0 | 0 | 0 | 0 | 0 |
| Ruprecht-Karls-Universität Heidelberg | Baden-Württemberg | 49,41095315 | 8,706640128 | 0 | 0 | 0 | 0 | 0 |
| Universität des Saarlandes Homburg | Saarland | 49,25531104 | 7,040940668 | 0 | 0 | 0 | 0 | 0 |
| Friedrich-Schiller Universität Jena | Thüringen | 50,92862196 | 11,58169463 | 0 | 0 | 0 | 0 | 0 |
| Christian-Albrechts-Universität Kiel | Schleswig-Holstein | 54,34361082 | 10,11499261 | 0 | 0 | 0 | 0 | 0 |
| Universität zu Köln | NRW | 50,92839842 | 6,929317882 | 0 | 0 | 0 | 0 | 0 |
| Universität Leipzig | Sachsen | 51,33894914 | 12,37867608 | 0 | 0 | 0 | 0 | 0 |
| Universität zu Lübeck | Schleswig-Holstein | 53,83442099 | 10,70369091 | 0 | 0 | 0 | 0 | 0 |
| Otto-von-Guericke-Universität Magdeburg | Sachsen-Anhalt | 52,14035109 | 11,6447011 | 0 | 0 | 0 | 0 | 0 |
| Johannes-Gutenberg-Universität Mainz | Rheinland-Pfalz | 49,99276442 | 8,241587384 | 0 | 0 | 0 | 0 | 0 |
| Medizinische Fakultät Mannheim der Universität Heidelberg | Baden-Württemberg | 49,50085392 | 8,493333347 | 0 | 0 | 0 | 0 | 0 |
| Philipps-Universität Marburg | Hessen | 50,81052134 | 8,773999213 | 0 | 0 | 0 | 0 | 0 |
| Ludwig-Maximilians-Universität München | Bayern | 48,15133363 | 11,57973215 | 0 | 0 | 0 | 0 | 0 |
| Technische Universität München | Bayern | 48,14906522 | 11,5683266 | 0 | 0 | 0 | 0 | 0 |
| Westfälische Wilhelms- Universität Münster | NRW | 51,96344192 | 7,613547468 | 0 | 0 | 0 | 0 | 0 |
| Carl-von-Ossietzky Universität Oldenburg | Niedersachsen | 53,14700353 | 8,183274201 | 0 | 0 | 0 | 0 | 0 |
| Universität Regensburg | Bayern | 48,9969171 | 12,09581898 | 0 | 0 | 0 | 0 | 0 |
| Universität Rostock | Mecklenburg-Vorpommern | 54,08539311 | 12,10114037 | 0 | 0 | 0 | 0 | 0 |
| Eberhard-Karls-Universität Tübingen | Baden-Württemberg | 48,52527152 | 9,057319784 | 0 | 0 | 0 | 0 | 0 |
| Universtität Ulm | Baden-Württemberg | 48,4235755 | 9,95517542 | 0 | 0 | 0 | 0 | 0 |
| Julius-Maximilians-Universität Würzburg | Bayern | 49,78836147 | 9,935281455 | 0 | 0 | 0 | 0 | 0 |

Scripts for Fig. 1-5

\# Set your working directory if needed

\# setwd("your/path/here")

################################################################################

###############################################################################

###############################################################################

#Figure 1 A

\# ==============================================================

\# 1) Load packages

\# ==============================================================

library(readxl)

library(dplyr)

library(tidyr)

library(ggplot2)

\# ==============================================================

\# 2) Read Excel file

\# ==============================================================

file <- "ratio_uro.xlsx"

df <- read_excel(file)

\# Ensure all relevant columns are numeric

num_cols <- setdiff(names(df), "Year")

df[num_cols] <- lapply(df[num_cols], as.numeric)

df$Year <- as.numeric(df$Year)

\# ==============================================================

\# 3) Convert to long format

\# ==============================================================

df_long <- df %>%

pivot_longer(

cols	= -Year,

names_to = "Category",

values_to = "Ratio"

)

\# Define category order

df_long$Category <- factor(

df_long$Category,

levels = c(

"Medical students",

"Specialised physicians",

"Leading positions",

"Chair holder"

)

)

\# ==============================================================

\# 4) Bar chart

\# ==============================================================

ggplot(df_long, aes(x = factor(Year), y = Ratio, fill = Category)) +

geom_col(

position = position_dodge(width = 0.8),

width	= 0.7

) +

geom_hline(

yintercept = 1,

linetype	= "dashed",

color	= "black"

) +

scale_fill_manual(

values = c(

"Medical students"	= "#595959",

"Specialised physicians" = "#4CAF50",

"Leading positions"	= "#2C7BB6",

"Chair holder"	= "#F1A340"

)

) +

scale_y_continuous(

limits = c(0, 2),

breaks = seq(0, 2, 0.25),

expand = c(0, 0)

) +

labs(

x	= "Year",

y	= "f:m Ratio",

fill = NULL

) +

theme_minimal(base_size = 14) +

theme(

panel.grid	= element_blank(),

axis.line	= element_line(color = "black"),

axis.ticks	= element_line(color = "black"),

axis.text	= element_text(color = "black"),

axis.title	= element_text(face = "bold"),

legend.position = "bottom"

)

################################################################################

###############################################################################

###############################################################################

#Figure 1 B

\# ==============================================================

\# 1) Load packages

\# ==============================================================

library(readxl)

library(dplyr)

library(tidyr)

library(ggplot2)

\# ==============================================================

\# 2) Read Excel file

\# ==============================================================

file <- "ratio_gyn.xlsx"

df <- read_excel(file)

\# Ensure all relevant columns are numeric

num_cols <- setdiff(names(df), "Year")

df[num_cols] <- lapply(df[num_cols], as.numeric)

df$Year <- as.numeric(df$Year)

\# ==============================================================

\# 3) Convert to long format

\# ==============================================================

df_long <- df %>%

pivot_longer(

cols	= -Year,

names_to = "Category",

values_to = "Ratio"

)

\# Define category order

df_long$Category <- factor(

df_long$Category,

levels = c(

"Medical students",

"Specialised physicians",

"Leading positions",

"Chair holder"

)

)

\# ==============================================================

\# 4) Bar chart

\# ==============================================================

ggplot(df_long, aes(x = factor(Year), y = Ratio, fill = Category)) +

geom_col(

position = position_dodge(width = 0.8),

width	= 0.7

) +

geom_hline(

yintercept = 1,

linetype	= "dashed",

color	= "black"

) +

scale_fill_manual(

values = c(

"Medical students"	= "#595959",

"Specialised physicians" = "#4CAF50",

"Leading positions"	= "#2C7BB6",

"Chair holder"	= "#F1A340"

)

) +

scale_y_continuous(

limits = c(0, 2.4),

breaks = seq(0, 2.4, 0.25),

expand = c(0, 0)

) +

labs(

x	= "Year",

y	= "f:m Ratio",

fill = NULL

) +

theme_minimal(base_size = 14) +

theme(

panel.grid	= element_blank(),

axis.line	= element_line(color = "black"),

axis.ticks	= element_line(color = "black"),

axis.text	= element_text(color = "black"),

axis.title	= element_text(face = "bold"),

legend.position = "bottom"

)

################################################################################

###############################################################################

###############################################################################

#Figure 2

\# ==============================================================

\# 1) Load packages

\# ==============================================================

library(readxl)

library(dplyr)

library(tidyr)

library(ggplot2)

library(grid)

\# --------------------------------------------------------------

\# 2) Function

\# --------------------------------------------------------------

make_index_plot <- function(file) {

df <- read_excel(file)

names(df) <- gsub("^X", "", names(df))

df$Department <- trimws(df$Department)

df$Index	<- trimws(df$Index)

year_cols <- grep("^\\d{4}$", names(df), value = TRUE)

df[year_cols] <- lapply(df[year_cols], as.numeric)

df_long <- df %>%

pivot_longer(

cols	= all_of(year_cols),

names_to = "Year",

values_to = "Value"

) %>%

mutate(

Year = as.numeric(Year),

LegendLabel = paste(Department, Index)

)

\# Fixed legend order

df_long$LegendLabel <- factor(

df_long$LegendLabel,

levels = c(

"OB/GYN Academic BG-Index",

"OB/GYN Clinical BG-Index",

"Urology Academic BG-Index",

"Urology Clinical BG-Index"

)

)

\# Colors by department

legend_colors <- c(

"OB/GYN Academic BG-Index"	= "#F1A340",

"OB/GYN Clinical BG-Index"	= "#F1A340",

"Urology Academic BG-Index" = "#2FB47C",

"Urology Clinical BG-Index" = "#2FB47C"

)

\# Line types by index type

legend_linetypes <- c(

"OB/GYN Academic BG-Index"	= "solid",

"OB/GYN Clinical BG-Index"	= "dashed",

"Urology Academic BG-Index" = "solid",

"Urology Clinical BG-Index" = "dashed"

)

ggplot(

df_long,

aes(

x	= Year,

y	= Value,

color	= LegendLabel,

linetype = LegendLabel

)

) +

geom_line(linewidth = 1.2) +

geom_hline(yintercept = 0, color = "black", linewidth = 0.4) +

scale_color_manual(values = legend_colors, drop = FALSE) +

scale_linetype_manual(values = legend_linetypes, drop = FALSE) +

scale_x_continuous(breaks = sort(unique(df_long$Year))) +

scale_y_continuous(

limits = c(0, 0.25),

breaks = seq(0, 0.25, 0.05),

expand = c(0, 0)

) +

labs(

x = "Year",

y = "BG Index",

color = NULL,

linetype = NULL

) +

theme_minimal(base_size = 14) +

theme(

panel.grid	= element_blank(),

axis.line.y	= element_line(color = "black"),

axis.text	= element_text(color = "black"),

axis.title	= element_text(face = "bold"),

legend.position = "top"

) +

guides(color = guide_legend(ncol = 2))

}

\# ==============================================================

\# 3) Plot

\# ==============================================================

plot_bg <- make_index_plot("BGindex.xlsx")

print(plot_bg)

################################################################################

###############################################################################

###############################################################################

#Figure 3

############################

\# GYN – Spatial Plot

############################

\# =========================================

\# 1) Load packages

\# =========================================

packages <- c(

"readr", "dplyr", "tidyr", "ggplot2",

"viridis", "sf", "rnaturalearth", "rnaturalearthdata"

)

to_install <- packages[!(packages %in% installed.packages()[, "Package"])]

if (length(to_install)) install.packages(to_install)

library(readr)

library(dplyr)

library(tidyr)

library(ggplot2)

library(viridis)

library(sf)

library(rnaturalearth)

library(rnaturalearthdata)

\# =========================================

\# 2) Read file

\# =========================================

file <- "gyn.txt"

data <- read_delim(

file,

delim = "\t",

locale = locale(encoding = "Latin1"),

show_col_types = FALSE

)

colnames(data) <- trimws(colnames(data))

\# =========================================

\# 3) Convert to long format + cleaning

\# =========================================

percentage_columns <- c(

"Anteil Frauen 2004",

"Anteil Frauen 2009",

"Anteil Frauen 2014",

"Anteil Frauen 2019",

"Anteil Frauen 2024"

)

data_long <- data %>%

pivot_longer(

cols	= all_of(percentage_columns),

names_to = "Year",

values_to = "Percentage"

) %>%

mutate(

Year	= as.integer(gsub("Anteil( der)? Frauen ?", "", Year)),

Percentage = as.numeric(Percentage),

Long	= as.numeric(Long),

Lat	= as.numeric(Lat)

) %>%

filter(!is.na(Long), !is.na(Lat), !is.na(Percentage))

\# =========================================

\# 4) Create fixed offsets for each location

\# =========================================

set.seed(123)

data_long <- data_long %>%

group_by(Long, Lat) %>%

mutate(

offset_x = runif(1, -0.05, 0.05),

offset_y = runif(1, -0.05, 0.05)

) %>%

ungroup() %>%

mutate(

Long_j = Long + offset_x,

Lat_j = Lat + offset_y

)

\# =========================================

\# 5) Sort: positive values plotted on top

\# =========================================

data_long <- data_long %>%

mutate(order = ifelse(Percentage > 0, 2, 1)) %>%

arrange(order)

\# =========================================

\# 6) Load map of Germany

\# =========================================

germany <- ne_countries(

scale	= "medium",

country	= "Germany",

returnclass = "sf"

)

\# =========================================

\# 7) Create plot

\# =========================================

ggplot(data_long) +

geom_sf(

data = germany,

fill = "gray95",

color = "gray70"

) +

geom_point(

aes(x = Long_j, y = Lat_j, fill = Percentage),

shape = 21,

color = "black",

size	= 2.5,

stroke = 0.5,

alpha = 0.9

) +

scale_fill_gradient(

low	= "yellow",

high	= "darkgreen",

limits = c(0, 100),

name	= "[%]"

) +

coord_sf(

xlim	= c(5, 15.5),

ylim	= c(47, 55),

expand = FALSE

) +

facet_wrap(~ Year) +

theme_void() +

theme(

legend.position = "right",

strip.text	= element_text(size = 14, face = "bold"),

plot.title	= element_text(size = 14, face = "bold", hjust = 0.5)

)

################################################################################

###############################################################################

###############################################################################

#Figure 4

############################

\# URO – Spatial Plot

############################

\# =========================================

\# 1) Load packages

\# =========================================

library(readr)

library(dplyr)

library(tidyr)

library(ggplot2)

library(viridis)

library(rnaturalearth)

library(rnaturalearthdata)

library(sf)

\# =========================================

\# 2) Read file

\# =========================================

filename <- "uro.txt"

if (!file.exists(filename)) {

stop(paste("File not found:", filename))

}

data <- read_delim(

filename,

delim = "\t",

locale = locale(encoding = "Latin1"),

show_col_types = FALSE

)

\# =========================================

\# 3) Clean column names

\# =========================================

colnames(data) <- trimws(colnames(data))

\# =========================================

\# 4) Define relevant percentage columns

\# =========================================

percentage_columns <- c(

"Anteil Frauen 2004",

"Anteil Frauen 2009",

"Anteil Frauen 2014",

"Anteil Frauen 2019",

"Anteil Frauen 2024"

)

\# =========================================

\# 5) Convert to long format + cleaning

\# =========================================

data_long <- data %>%

pivot_longer(

cols	= all_of(percentage_columns),

names_to = "Year",

values_to = "Percentage"

) %>%

mutate(

Year	= as.integer(gsub("Anteil Frauen ", "", Year)),

Percentage = as.numeric(Percentage),

Long	= as.numeric(Long),

Lat	= as.numeric(Lat)

) %>%

filter(!is.na(Long), !is.na(Lat), !is.na(Percentage))

\# =========================================

\# 6) Create fixed offsets for each location

\# =========================================

set.seed(123)

data_long <- data_long %>%

group_by(Long, Lat) %>%

mutate(

offset_x = runif(1, -0.05, 0.05),

offset_y = runif(1, -0.05, 0.05)

) %>%

ungroup() %>%

mutate(

Long_j = Long + offset_x,

Lat_j = Lat + offset_y

)

\# =========================================

\# 7) Load map of Germany

\# =========================================

germany <- ne_countries(

scale	= "medium",

country	= "Germany",

returnclass = "sf"

)

\# =========================================

\# 8) Create plot

\# =========================================

ggplot(data_long) +

geom_sf(

data = germany,

fill = "gray95",

color = "gray70"

) +

geom_point(

aes(x = Long_j, y = Lat_j, fill = Percentage),

shape = 21,

color = "black",

size	= 2.5,

stroke = 0.5,

alpha = 0.9

) +

scale_fill_gradient(

low	= "yellow",

high	= "darkgreen",

limits = c(0, 100),

name	= "[%]"

) +

coord_sf(

xlim	= c(5, 15.5),

ylim	= c(47, 55),

expand = FALSE

) +

facet_wrap(~ Year) +

theme_void() +

theme(

legend.position = "right",

strip.text	= element_text(size = 14, face = "bold"),

plot.title	= element_text(size = 14, face = "bold", hjust = 0.5)

)

################################################################################

###############################################################################

###############################################################################

#Figure 5

\# ==============================================================

\# SUMMARY GRAPH

\# ==============================================================

\# ==============================================================

\# 1) Load packages

\# ==============================================================

packages <- c(

"readr", "dplyr", "tidyr", "ggplot2",

"sf", "rnaturalearth", "rnaturalearthdata"

)

to_install <- packages[!(packages %in% installed.packages()[, "Package"])]

if (length(to_install)) install.packages(to_install)

library(readr)

library(dplyr)

library(tidyr)

library(ggplot2)

library(sf)

library(rnaturalearth)

library(rnaturalearthdata)

\# ==============================================================

\# 2) Helper function: standardize column names

\# ==============================================================

standardize_columns <- function(df) {

colnames(df) <- gsub("Anteil der Frauen", "Anteil Frauen", colnames(df))

colnames(df) <- trimws(colnames(df))

return(df)

}

\# ==============================================================

\# 3) Define percentage columns

\# ==============================================================

percentage_cols <- c(

"Anteil Frauen 2004",

"Anteil Frauen 2009",

"Anteil Frauen 2014",

"Anteil Frauen 2019",

"Anteil Frauen 2024"

)

\# ==============================================================

\# 4) Load and process gynaecology data

\# ==============================================================

gyn <- read_delim(

"gyn.txt",

delim = "\t",

locale = locale(encoding = "Latin1")

)

gyn <- standardize_columns(gyn)

gyn <- gyn %>%

mutate(across(all_of(percentage_cols), ~ as.numeric(gsub(",", ".", .)))) %>%

rowwise() %>%

mutate(has_woman_gyn = any(c_across(all_of(percentage_cols)) > 0, na.rm = TRUE)) %>%

ungroup() %>%

select(Uni = Universität, Long, Lat, has_woman_gyn)

\# ==============================================================

\# 5) Load and process urology data

\# ==============================================================

uro <- read_delim(

"uro.txt",

delim = "\t",

locale = locale(encoding = "Latin1")

)

uro <- standardize_columns(uro)

uro <- uro %>%

mutate(across(all_of(percentage_cols), ~ as.numeric(gsub(",", ".", .)))) %>%

rowwise() %>%

mutate(has_woman_uro = any(c_across(all_of(percentage_cols)) > 0, na.rm = TRUE)) %>%

ungroup() %>%

select(Uni = Universität, Long, Lat, has_woman_uro)

\# ==============================================================

\# 6) Merge data and assign category

\# ==============================================================

full <- full_join(gyn, uro, by = c("Uni", "Long", "Lat")) %>%

mutate(

has_woman_gyn = ifelse(is.na(has_woman_gyn), FALSE, has_woman_gyn),

has_woman_uro = ifelse(is.na(has_woman_uro), FALSE, has_woman_uro),

Category = case_when(

has_woman_gyn & has_woman_uro ~ "Both fields",

has_woman_gyn & !has_woman_uro ~ "Only Gynaecology",

!has_woman_gyn & has_woman_uro ~ "Only Urology",

TRUE	~ "None"

),

order = case_when(

Category == "None"	~ 1,

Category == "Only Urology"	~ 2,

Category == "Only Gynaecology" ~ 3,

Category == "Both fields"	~ 4

)

) %>%

arrange(order)

\# ==============================================================

\# 7) Load map of Germany

\# ==============================================================

germany <- ne_countries(

scale = "medium",

country = "Germany",

returnclass = "sf"

)

\# ==============================================================

\# 8) Define updated color palette

\# ==============================================================

colors <- c(

"Both fields"	= "#b2182b", # dark red/pink

"Only Gynaecology" = "#ffa500", # orange

"Only Urology"	= "#006837", # green

"None"	= "#bdbdbd"	# medium gray

)

\# ==============================================================

\# 9) Plot the map

\# ==============================================================

ggplot() +

geom_sf(data = germany, fill = "gray95", color = "gray70") +

geom_point(

data = full,

aes(x = Long, y = Lat, fill = Category),

shape = 21,

size = 5.0,

color = "black",

stroke = 0.4,

alpha = 0.95,

position = position_jitter(width = 0.05, height = 0.05)

) +

scale_fill_manual(

values = colors,

name = NULL,

breaks = c("Both fields", "Only Gynaecology", "Only Urology", "None")

) +

coord_sf(

xlim = c(5, 15.5),

ylim = c(47, 55),

expand = FALSE

) +

theme_void() +

theme(

legend.position = "right",

legend.title = element_text(size = 14, face = "bold"),

legend.text = element_text(size = 12),

legend.key.size = unit(0.8, "cm"),

plot.title = element_text(hjust = 0.5, size = 18, face = "bold")

)

################################################################################

###############################################################################

###############################################################################

#Figure 6

#######################################################################

\# Federal state GYN

\# ==============================================================

\# 1) Load packages

\# ==============================================================

library(readr)

library(dplyr)

library(tidyr)

library(ggplot2)

library(viridis)

library(sf)

library(rgeoboundaries)

\# ==============================================================

\# 2) Read file

\# ==============================================================

file <- "gyn.txt"

dat <- read_delim(file, delim = "\t", locale = locale(encoding = "Latin1")) %>%

mutate(across(everything(), ~ trimws(as.character(.)))) # Remove whitespace

\# ==============================================================

\# 3) Convert to long format + status (positive/negative)

\# ==============================================================

pct_cols <- c(

"Anteil Frauen 2004",

"Anteil Frauen 2009",

"Anteil Frauen 2014",

"Anteil Frauen 2019",

"Anteil Frauen 2024"

)

dat_long <- dat %>%

pivot_longer(

cols = all_of(pct_cols),

names_to = "Year",

values_to = "Percentage"

) %>%

mutate(

Year = as.integer(gsub("Anteil( der)? Frauen ?", "", Year)),

Percentage = as.numeric(Percentage),

Status = ifelse(Percentage > 0, "positive", "negative")

) %>%

filter(!is.na(Long), !is.na(Lat), !is.na(Percentage))

\# ==============================================================

\# 4) Count positive locations by federal state and year

\# ==============================================================

dat_long$Bundesland <- trimws(dat_long$Bundesland)

count_df <- dat_long %>%

filter(Status == "positive") %>%

count(Bundesland, Year, name = "n_positive")

\# ==============================================================

\# 5) Mapping: German names as used in `states_sf`

\# ==============================================================

name_map <- c(

"NRW"	= "Nordrhein-Westfalen",

"Bayern"	= "Bayern",

"Berlin"	= "Berlin",

"Sachsen"	= "Sachsen",

"Hessen"	= "Hessen",

"Baden-Württemberg"	= "Baden-Württemberg",

"Niedersachsen"	= "Niedersachsen",

"Mecklenburg-Vorpommern" = "Mecklenburg-Vorpommern",

"Sachsen-Anhalt"	= "Sachsen-Anhalt",

"Hamburg"	= "Hamburg",

"Saarland"	= "Saarland",

"Thüringen"	= "Thüringen",

"Schleswig-Holstein"	= "Schleswig-Holstein",

"Rheinland-Pfalz"	= "Rheinland-Pfalz"

)

count_df$shapeName <- name_map[count_df$Bundesland]

\# ==============================================================

\# 6) Load geometry

\# ==============================================================

states_sf <- geoboundaries("Germany", adm_lvl = "ADM1")

states_sf$shapeName <- trimws(states_sf$shapeName)

\# ==============================================================

\# 7) Create all combinations of shapeName × year

\# ==============================================================

all_years <- sort(unique(dat_long$Year))

all_shapes <- sort(unique(states_sf$shapeName))

template <- expand.grid(

Year = all_years,

shapeName = all_shapes,

stringsAsFactors = FALSE

)

\# ==============================================================

\# 8) Fill count values (NA → 0) and join with geometry

\# ==============================================================

count_complete <- left_join(template, count_df, by = c("Year", "shapeName")) %>%

mutate(n_positive = ifelse(is.na(n_positive), 0, n_positive))

map_df <- left_join(states_sf, count_complete, by = "shapeName")

\# ==============================================================

\# 9) Define color scale and draw map

\# ==============================================================

\# Discrete color palette:

\# 0 = gray, 1 = light blue, 2-3 = dark blue

custom_pal <- c(

"#d9d9d9", # 0

"#9ecae1", # 1

"#2171b5"	# 2-3

)

ggplot(map_df) +

geom_sf(aes(fill = n_positive), color = "white", linewidth = 0.2) +

scale_fill_gradientn(

colours = custom_pal,

limits = c(0, 3),

breaks = c(0, 1, 2, 3),

labels = c("0", "1", "2", "3"),

name	= "Number per state"

) +

facet_wrap(~ Year) +

theme_void() +

theme(

legend.position = "bottom",

legend.key.width = unit(2, "cm"),

strip.text = element_text(size = 12, face = "bold"),

plot.title = element_text(size = 16, face = "bold", hjust = 0.5)

)

################################################################################

###############################################################################

###############################################################################

#Figure 7

#################################################################

#################################################################

#################################################################

\# FEDERAL STATE

\# ==============================================================

\# 1) Load packages

\# ==============================================================

library(readr)

library(dplyr)

library(tidyr)

library(ggplot2)

library(viridis)

library(sf)

library(rgeoboundaries)

\# ==============================================================

\# 2) Read file

\# ==============================================================

file <- "uro.txt"

dat <- read_delim(file, delim = "\t", locale = locale(encoding = "Latin1")) %>%

mutate(across(everything(), ~ trimws(as.character(.)))) # Remove whitespace

\# ==============================================================

\# 3) Convert to long format + status (positive/negative)

\# ==============================================================

pct_cols <- c(

"Anteil Frauen 2004",

"Anteil Frauen 2009",

"Anteil Frauen 2014",

"Anteil Frauen 2019",

"Anteil Frauen 2024"

)

dat_long <- dat %>%

pivot_longer(

cols = all_of(pct_cols),

names_to = "Year",

values_to = "Percentage"

) %>%

mutate(

Year = as.integer(gsub("Anteil( der)? Frauen ?", "", Year)),

Percentage = as.numeric(Percentage),

Status = ifelse(Percentage > 0, "positive", "negative")

) %>%

filter(!is.na(Long), !is.na(Lat), !is.na(Percentage))

\# ==============================================================

\# 4) Count positive locations by federal state and year

\# ==============================================================

dat_long$Bundesland <- trimws(dat_long$Bundesland)

count_df <- dat_long %>%

filter(Status == "positive") %>%

count(Bundesland, Year, name = "n_positive")

\# ==============================================================

\# 5) Mapping: German names as used in `states_sf`

\# ==============================================================

name_map <- c(

"NRW"	= "Nordrhein-Westfalen",

"Bayern"	= "Bayern",

"Berlin"	= "Berlin",

"Sachsen"	= "Sachsen",

"Hessen"	= "Hessen",

"Baden-Württemberg"	= "Baden-Württemberg",

"Niedersachsen"	= "Niedersachsen",

"Mecklenburg-Vorpommern" = "Mecklenburg-Vorpommern",

"Sachsen-Anhalt"	= "Sachsen-Anhalt",

"Hamburg"	= "Hamburg",

"Saarland"	= "Saarland",

"Thüringen"	= "Thüringen",

"Schleswig-Holstein"	= "Schleswig-Holstein",

"Rheinland-Pfalz"	= "Rheinland-Pfalz"

)

count_df$shapeName <- name_map[count_df$Bundesland]

\# ==============================================================

\# 6) Load geometry

\# ==============================================================

states_sf <- geoboundaries("Germany", adm_lvl = "ADM1")

states_sf$shapeName <- trimws(states_sf$shapeName)

\# ==============================================================

\# 7) Create all combinations of shapeName × year

\# ==============================================================

all_years <- sort(unique(dat_long$Year))

all_shapes <- sort(unique(states_sf$shapeName))

template <- expand.grid(

Year = all_years,

shapeName = all_shapes,

stringsAsFactors = FALSE

)

\# ==============================================================

\# 8) Fill count values (NA → 0) and join with geometry

\# ==============================================================

count_complete <- left_join(template, count_df, by = c("Year", "shapeName")) %>%

mutate(n_positive = ifelse(is.na(n_positive), 0, n_positive))

map_df <- left_join(states_sf, count_complete, by = "shapeName")

\# ==============================================================

\# 9) Define color scale and draw map

\# ==============================================================

\# Discrete color palette:

\# 0 = gray, 1 = light blue, 2-3 = dark blue

custom_pal <- c(

"#d9d9d9", # 0

"#9ecae1", # 1

"#2171b5"	# 2-3

)

ggplot(map_df) +

geom_sf(aes(fill = n_positive), color = "white", linewidth = 0.2) +

scale_fill_gradientn(

colours = custom_pal,

limits = c(0, 3),

breaks = c(0, 1, 2, 3),

labels = c("0", "1", "2", "3"),

name	= "Number per state"

) +

facet_wrap(~ Year) +

theme_void() +

theme(

legend.position = "bottom",

legend.key.width = unit(2, "cm"),

strip.text = element_text(size = 12, face = "bold"),

plot.title = element_text(size = 16, face = "bold", hjust = 0.5)

)
