# Supplementary material for: Female career advancement in obstetrics/gynecology, and urology: a spatio-temporal comparative study over the last 2 decades in Germany
Source: Arch Gynecol Obstet. 2026 Sep 3;313(1):264. doi: 10.1007/s00404-026-08511-2 (PMC13541887; doi:10.1007/s00404-026-08511-2)
Supplement: Supplementary file 2 [file 404_2026_8511_MOESM2_ESM.docx]

# Supplementary table, supplementary information

**Fig. S1** Spatial analysis by federal states across Germany of the number of female chairholders in the department of obstetrics (OB) and gynecology (GYN) (2004-2024)

**Fig. S2** Spatial analysis by federal states across Germany of the number of female chairholders in the department of urology (2004-2024)

**Table S1** Gender of chair holders of 43 German medical faculties (2004-2024) in the fields of obstetrics (OB)/ gynecology (GYN) and urology. The chair holders' gender is described as female (F) (for better visualisation highlighted in grey) or male (M), in case of multiple chair holders the amount of people is noted with the number of chair holders, followed by the gender.

|  |  |  | OB/GYN |  |  | Urology | | | | |
| --- | --- | --- | --- | --- | --- | --- | --- | --- | --- | --- |
| Medical faculty | 2004 | 2009 | 2014 | 2019 | 2024 | 2004 | 2009 | 2014 | 2019 | 2024 |
| RWTH Aachen | M | M | M | M | M | M | M | M | M | M |
| Universität Augsburg | - | - | - | M | M | - | - | - | F | F |
| Charité Berlin | 2M | 2M | 2M | 2M | 2M | M | M | M | M | M |
| Universität Bielefeld | - | - | - | - | F | - | - | - | - | M |
| Ruhr-Universität Bochum | 2M | 2M | M | M | M | M | M | M | M | M |
| Friedrich-Wilhelms-Universität Bonn | 3M | 3M | 3M | 3M,F | 2M,2F | M | M | M | M | M |
| Medizinische Hochschule Brandenburg | - | - | - | F | - | - | - | - | M | M |
| Technische Universität Dresden | M | M | M | F | F | M | M | M | M | M |
| Technische Universität Dresden / Chemnitz | M | M | M | M | M | M | M | M | M | M |
| Heinrich-Heine-Universität Düsseldorf | M | M | F | F | F | M | M | M | M | M |
| Friedrich-Alexander-Universität Erlangen-Nürnberg | M | M | M | M | M | M | M | M | M | M |
| Friedrich-Alexander-Universität Erlangen-Nürnberg / Bayreuth | M | M | M | M | M | M | M | M | M | M |
| Universität Duisburg-Essen | M | M | M | M | M | M | M | M | M | M |
| Johann-Wolfgang-Goethe-Universität Frankfurt am Main | M | M | M | M | M | M | M | M | M | M |
| Albert-Ludwigs-Universität Freiburg | M | M | M | M | M | M | M | M | M | M |
| Justus-Liebig-Universität Gießen | M | M | M | M | M | M | M | M | M | M |
| Georg-August-Universität Göttingen | M | M | M | M | F | M | M | M | M | M |
| Ernst-Moritz-Arndt-Universität Greifswald | M | M | M | M | M | M | M | M | M | M |
| Martin-Luther-Universität Halle-Wittenberg | M | M | M | M | M | M | M | M | M | M |
| Universität Hamburg-Eppendorf | M | M | M | F | F | M | F | F | F | F |
| Medizinische Hochschule Hannover | M | M | M | M | M | M | M | M | M | M |
| Ruprecht-Karls-Universität Heidelberg | M | M | M | M | M | M | M | M | M | M |
| Ruprecht-Karls-Universität Heidelberg / Mannheim | M | M | M | M | M | M | M | M | M | M |
| Friedrich-Schiller-Universität Jena | 2M | 2M | 2M | 2M | 2M | M | M | M | M | M |
| Christian-Albrechts-Universität Kiel | M | M | M | M | M | M | M | M | M | M |
| Universität Köln | M | M | M | M | M | M | M | M | M | M |
| Universität Leipzig | M | M | M | F | F | M | M | M | M | M |
| Universität Lübeck | M | M | M | M | M | M | M | M | M | M |
| Otto-von-Guericke-Universität Magdeburg | M | M | M | M | M | M | - | M | M | M |
| Johannes-Gutenberg-Universität Mainz | M | M | - | F | F | M | M | M | M | M |
| Johannes-Gutenberg-Universität Mainz – Medizincampus Trier | M | M | M | M | M | M | M | M | M | M |
| Philipps-Universität Marburg | M | M | M | M | M | M | M | M | M | M |
| Ludwig-Maximilians-Universität München | M | M | M | M | M | M | M | M | M | M |
| TU München | F | F | F | F | F | M | M | M | M | M |
| Westfälische Wilhelms-Universität Münster | M | M | M | M | M | M | M | M | M | M |
| Carl-von-Ossietzky-Universität Oldenburg | - | - | - | M | M | - | - | - | - | - |
| Universität Regensburg | F | F | F | F | F | M | M | M | M | M |
| Universität Regensburg MCN | M | M | M | M | M | M | M | M | M | M |
| Universität Rostock | - | M | M | M | M | M | M | M | M | M |
| Universität des Saarlandes Saarbrücken | M | M | M | M | M | M | M | M | M | M |
| Eberhard-Karls-Universität Tübingen | M | M | M | M | F | M | M | M | M | M |
| Universität Ulm | M | M | M | M | M | M | M | M | M | M |
| Julius-Maximilians-Universität Würzburg | M | M | M | M | M | M | M | M | M | M |
